# Physiological, Biochemical, and Structural Bioinformatic Analysis of the Multiple Inositol Dehydrogenases from Corynebacterium glutamicum

**DOI:** 10.1128/spectrum.01950-22

**Published:** 2022-09-12

**Authors:** Paul Ramp, Christopher Pfleger, Jonas Dittrich, Christina Mack, Holger Gohlke, Michael Bott

**Affiliations:** a IBG-1: Biotechnology, Institute of Bio- and Geosciences, Forschungszentrum Jülich, Jülich, Germany; b The Bioeconomy Science Center (BioSC), Forschungszentrum Jülich, Jülich, Germany; c Institut für Pharmazeutische und Medizinische Chemie, Heinrich-Heine-Universität Düsseldorf, Düsseldorf, Germany; d John von Neumann Institute for Computing (NIC), Forschungszentrum Jülich GmbH, Jülich, Germany; e Jülich Supercomputing Centre (JSC), Forschungszentrum Jülich GmbH, Jülich, Germany; f Institute of Biological Information Processing (IBI-7: Structural Biochemistry), Forschungszentrum Jülich GmbH, Jülich, Germany; g Institute of Bio- and Geosciences (IBG-4: Bioinformatics), Forschungszentrum Jülich GmbH, Jülich, Germany; University of Southern Denmark

**Keywords:** inositol metabolism, *myo*-inositol, *scyllo*-inositol, d-*chiro*-inositol, enzyme kinetics, structural models, molecular docking, *Corynebacterium glutamicum*, inositols, inositol dehydrogenase

## Abstract

Inositols (cyclohexanehexols) comprise nine isomeric cyclic sugar alcohols, several of which occur in all domains of life with various functions. Many bacteria can utilize inositols as carbon and energy sources via a specific pathway involving inositol dehydrogenases (IDHs) as the first step of catabolism. The microbial cell factory Corynebacterium glutamicum can grow with *myo*-inositol as a sole carbon source. Interestingly, this species encodes seven potential IDHs, raising the question of the reason for this multiplicity. We therefore investigated the seven IDHs to determine their function, activity, and selectivity toward the biologically most important isomers *myo*-, *scyllo*-, and d-*chiro*-inositol. We created an ΔIDH strain lacking all seven IDH genes, which could not grow on the three inositols. *scyllo*- and d*-chiro*-inositol were identified as novel growth substrates of C. glutamicum. Complementation experiments showed that only four of the seven IDHs (IolG, OxiB, OxiD, and OxiE) enabled growth of the ΔIDH strain on two of the three inositols. The kinetics of the four purified enzymes agreed with the complementation results. IolG and OxiD are NAD^+^-dependent IDHs accepting *myo*- and d*-chiro*-inositol but not *scyllo*-inositol. OxiB is an NAD^+^-dependent *myo*-IDH with a weak activity also for *scyllo*-inositol but not for d-*chiro*-inositol. OxiE on the other hand is an NAD^+^-dependent *scyllo*-IDH showing also good activity for *myo*-inositol and a very weak activity for d*-chiro*-inositol. Structural models, molecular docking experiments, and sequence alignments enabled the identification of the substrate binding sites of the active IDHs and of residues allowing predictions on the substrate specificity.

**IMPORTANCE** *myo*-, *scyllo*-, and d-*chiro*-inositol are C_6_ cyclic sugar alcohols with various biological functions, which also serve as carbon sources for microbes. Inositol catabolism starts with an oxidation to keto-inositols catalyzed by inositol dehydrogenases (IDHs). The soil bacterium C. glutamicum encodes seven potential IDHs. Using a combination of microbiological, biochemical, and modeling approaches, we analyzed the function of these enzymes and identified four IDHs involved in the catabolism of inositols. They possess distinct substrate preferences for the three isomers, and modeling and sequence alignments allowed the identification of residues important for substrate specificity. Our results expand the knowledge of bacterial inositol metabolism and provide an important basis for the rational development of producer strains for these valuable inositols, which show pharmacological activities against, e.g., Alzheimer’s disease, polycystic ovarian syndrome, or type II diabetes.

## INTRODUCTION

Inositols (cyclohexanehexols) comprise a group of nine isomeric forms of C_6_-sugar alcohols having a cyclic structure formed by the six carbon atoms, each linked to a hydroxyl group. Depending on the orientation of the hydroxyl groups, nine isomers are possible, termed *myo*-, *scyllo*-, *epi*-, *allo*-, *muco*-, *neo*-, d*-chiro*, l-*chiro*-, and *cis*-inositol, all of which except the last occur in nature (1, 2). *myo*-Inositol (MI) is the predominant isomer used in biology and occurs in all kingdoms of life (3). It is synthesized from glucose 6-phosphate, which is converted by inositol 1-phosphate synthase (Ino1) to *myo*-inositol 1-phosphate followed by dephosphorylation to MI by an inositol monophosphatase (4–6). The other naturally occurring isomers are known or assumed to be derived from MI via epimerization (7, 8).

Numerous biological functions have been identified for inositols. For example, MI-containing phospholipids are constituents of the membranes of many archaea and all eukaryotes (3). Also, *scyllo*-inositol (SI) and d*-chiro*-inositol (DCI) were identified in lipids in some plant species (9, 10). Polyphosphorylated inositols (IP_1–3_) are key components of eukaryotic signaling pathways (3, 11), and MI hexakisphosphate (IP_6_), also known as phytic acid, is an abundant plant constituent serving as the main storage form of phosphate in seeds (12). In the bacterial kingdom, inositols play a prominent role, particularly in *Actinobacteria*. In this large phylum, MI is one of the precursors for the synthesis of mycothiol, a metabolite substituting for glutathione (13), and a precursor of phosphatidylinositol, an abundant phospholipid in the cytoplasmic membrane and the precursor of more complex lipids of the cell envelope such as phosphatidylinositol mannosides, lipomannan, and lipoarabinomannan (14).

Many bacteria are able to utilize MI as a carbon and energy source, such as Klebsiella aerogenes (15), Rhizobium leguminosarum (16), Bacillus subtilis (17, 18), Sinorhizobium meliloti (19, 20), Paracoccus laeviglucosivorans (21, 22), Legionella pneumophila (23), or Thermotoga maritima (24). After uptake via specific inositol transporters, MI is first oxidized by an inositol dehydrogenase (IDH) to yield the intermediate 2-keto-*myo*-inositol (2KMI), which is dehydrated to 3D-(3,5/4)-trihydroxycyclohexane-1,2-dione (THcHDO) by a 2KMI dehydratase (25, 26). This intermediate is converted in subsequent steps to dihydroxyacetone phosphate, acetyl coenzyme A (acetyl-CoA), and CO_2_ (15). The genes encoding the responsible enzymes are organized in large operons (27–29), which are usually regulated by a repressor called IolR that dissociates from its operator and enables gene expression when it forms a complex with intermediates of MI catabolism (30, 31).

**FIG 1:**
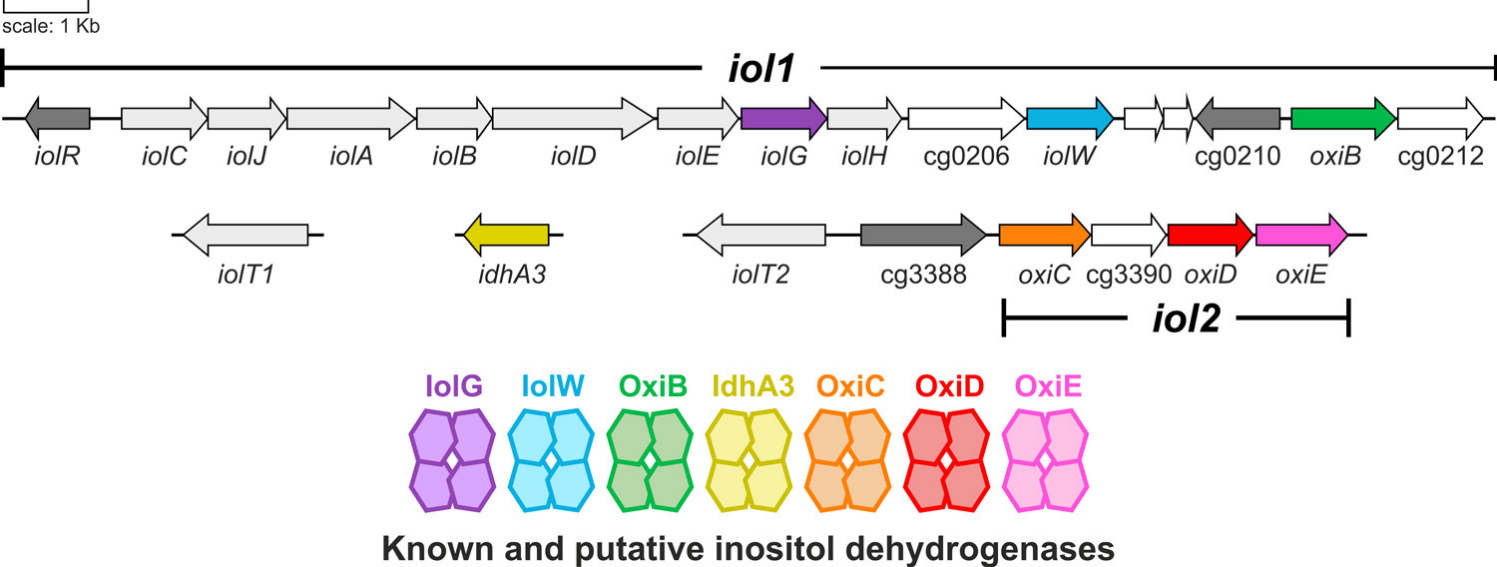
Organization of C. glutamicum genes involved in inositol transport and metabolism with the seven different IDH genes highlighted in color. The gene clusters *iol1* and *iol2* comprising genes involved in inositol metabolism are indicated.

Corynebacterium glutamicum is a soil-dwelling Gram-positive actinobacterium that is used as an industrial cell factory, in particular for large-scale production of l-glutamate and l-lysine (32–34). It can grow with MI as the sole carbon source (35). During growth on MI, more than 20 genes showed increased expression, most of which were located in two clusters on the genome. Cluster *iol1* (Fig. 1) contains 16 genes, which include a putative operon comprising 10 genes (cg0197 to cg0207), including those for the seven enzymes assumed to be responsible for MI conversion to dihydroxyacetone, acetyl-CoA, and CO_2_. Whereas many genes of cluster *iol1*, such as *iolD*, are essential for growth on MI, the genes of cluster *iol2* are dispensable (35). Two secondary transporters for MI uptake were identified in C. glutamicum, called IolT1 and IolT2 (35). In the absence of MI, expression of the genes involved in MI transport and degradation was shown to be repressed by the GntR-type transcriptional regulator IolR (36). C. glutamicum not only is able to degrade MI but also has the intrinsic capability to synthesize MI via MI phosphate synthase (Ino1, Cg3323) and MI phosphate monophosphatase (ImpA, Cg2298) (37). Expression of the *ino1* gene is activated by the LacI-type transcriptional regulator IpsA in response to the cytoplasmic MI concentration. When sufficient MI is present, it binds to IpsA and abolishes activation of *ino1* expression (38).

In many inositol-degrading bacteria, multiple paralogous genes annotated or shown to encode IDHs were identified (39–41), which, in the case of B. subtilis, for example, enable growth not only on MI but also on SI and DCI (42, 43). In C. glutamicum, seven potential inositol oxidoreductases were annotated, three in cluster *iol1* (IolG, IolW, and OxiB), three in cluster *iol2* (OxiC, OxiD, and OxiE), and another one elsewhere in the genome (IdhA3) (Fig. 1). This work aimed at a detailed characterization of the IDHs of C. glutamicum. Using a ΔIDH strain lacking the genes for all seven potential IDHs as a host for overexpression of the seven genes individually, we could show that four of the seven IDHs enable growth with MI, SI, or DCI (see Fig. S1 in the supplemental material) as the sole carbon and energy source. Biochemical characterization of the four purified enzymes revealed different activity profiles for the tested inositol isomers. We used structural modeling and molecular docking to elucidate the molecular basis responsible for the various substrate specificities of the four enzymes that may be helpful in predicting the substrate specificity of yet-uncharacterized inositol dehydrogenases in other organisms.

## RESULTS

### Growth on different inositols.

**FIG 2:**
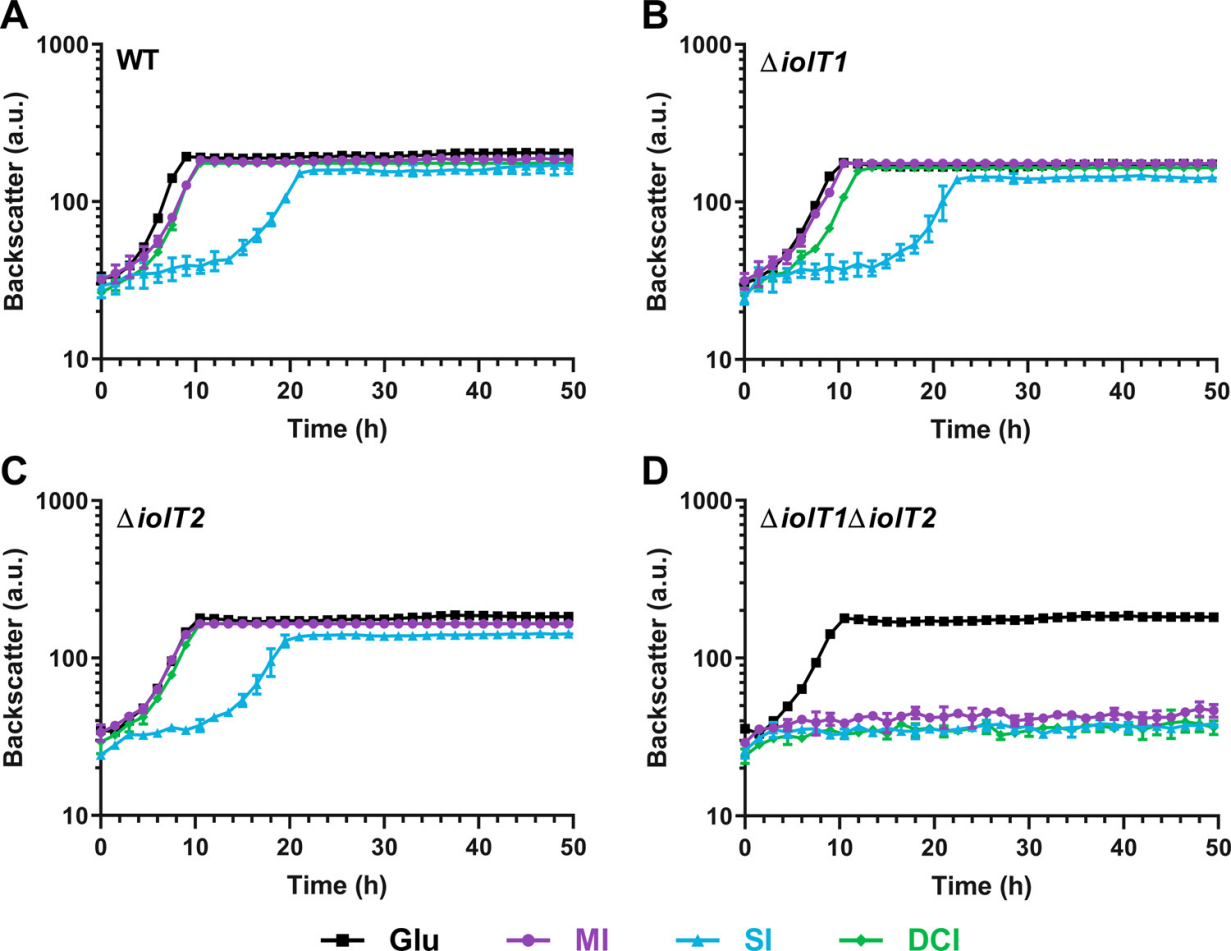
Growth of the C. glutamicum strains MB001(DE3) (A), ATCC 13032 Δ*iolT1* (B), ATCC 13032 Δ*iolT2* (C), and ATCC 13032 Δ*iolT1*Δ*iolT2* (D) on different inositols compared to glucose. The strains were cultivated in a BioLector system using CGXII minimal medium supplemented with glucose, MI, SI, or DCI at 10 g/L. The cultures were incubated for 48 h at 30°C, 1,200 rpm, and 85% humidity. Mean values and standard deviations for three biological replicates are shown. a.u., arbitrary units.

C. glutamicum can grow in minimal medium with MI as the sole carbon and energy source (35). The IDH IolG was shown to be important for growth on MI, as inactivation of *iolG* led to a reduced growth rate. Additional deletion of the gene cluster comprising *oxiC*-cg3390-*oxiD*-*oxiE* abolished the growth on MI, suggesting redundant MI dehydrogenase activities in C. glutamicum (35). To determine the potential of C. glutamicum to utilize further inositols besides MI for growth, we cultivated C. glutamicum MB001(DE3) in CGXII medium with either glucose, MI, SI, or DCI as the sole carbon and energy source using a BioLector microcultivation system. Growth was monitored by measuring backscatter at 620 nm over a time period of 48 h. This experiment showed that C. glutamicum is able to grow not only with MI but also with DCI and SI (Fig. 2A). The growth rate (μ) on MI (0.42 h^−1^) and DCI (0.42 h^−1^) was comparable to that on glucose (0.46 h^−1^), while the growth rate on SI was lower (0.26 h^−1^).

C. glutamicum possesses the two inositol transporters IolT1 and IolT2, both contributing to the uptake of MI (35). To test if DCI and SI enter the cells the same way, we analyzed the growth of the *ΔiolT1*, *ΔiolT2*, and *ΔiolT1ΔiolT2* transporter deletion mutant strains on glucose, MI, SI, and DCI. Indeed, both transporters contributed to the uptake of all tested inositols (Fig. 2B to D). With MI as the carbon source, the Δ*iolT1* and Δ*iolT2* strains showed comparable growth rates (0.42 h^−1^). In the case of DCI and SI, the Δ*iolT1* strain grew slightly slower (DCI, 0.38 h^−1^; SI, 0.24 h^−1^) than the Δ*iolT2* strain (DCI, 0.41 h^−1^; SI, 0.26 h^−1^), suggesting that IolT1 has a higher activity for DCI and SI uptake than IolT2 (Fig. 2B and C). Deletion of both *iolT1* and *iolT2* abolished growth on each of the three inositols completely (Fig. 2D), indicating that C. glutamicum does not possess an additional transporter for the uptake of inositols.

As an efficient approach for investigating the role of the seven annotated IDHs of C. glutamicum for growth on MI, SI, and DCI, we constructed the C. glutamicum ΔIDH strain, in which all seven IDH genes and the putative sugar phosphate isomerase gene cg3390, which is part of the *oxiC-*cg3390-*oxiD*-*oxiE* operon, were deleted (Fig. 1). C. glutamicum ΔIDH was transformed with pMKEx2-based expression plasmids encoding one of the seven IDHs and tested for growth on the different inositols. The successful synthesis of the individual IDH proteins was confirmed by SDS-PAGE (Fig. S2). As controls, the parent strain C. glutamicum MB001(DE3) and the ΔIDH strain were transformed with pMKEx2-*eyfp*. Target gene expression was induced by adding 20 μM isopropyl-β-d-thiogalactopyranoside (IPTG) to the second, overnight preculture and the main culture to enable an immediate start of growth.

**FIG 3:**
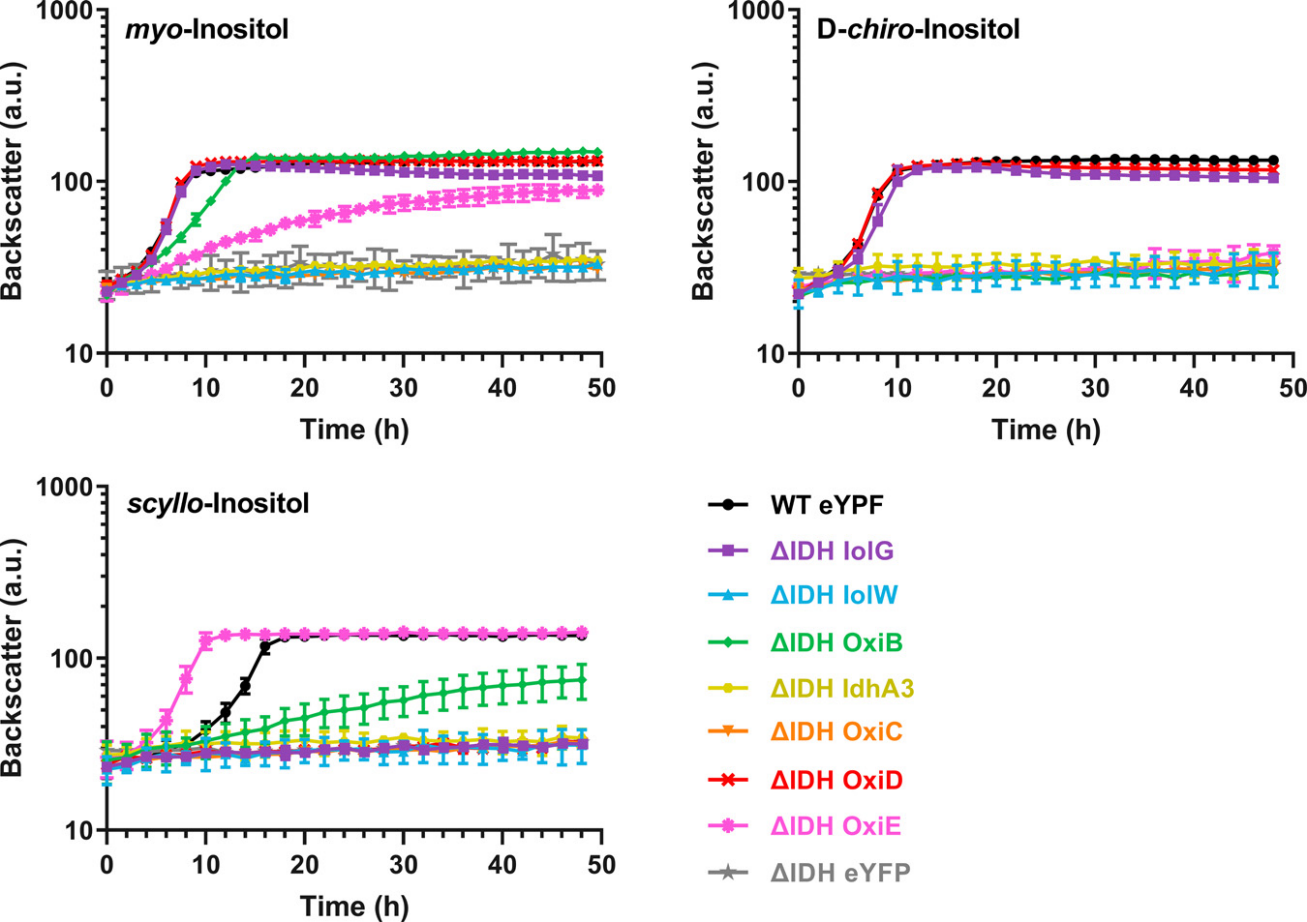
Growth on MI, SI, and DCI of C. glutamicum ΔIDH expressing one of the seven IDH genes or as negative-control enhanced yellow fluorescent protein (*eyfp*) using the corresponding pMKEx2-based plasmids. C. glutamicum MB001(DE3) transformed with pMKEx2-*eyfp* was used as a positive control. The strains were cultivated in a BioLector cultivation system for 48 h at 30°C, 1,200 rpm, and 85% humidity in CGXII minimal medium supplemented with 10 g/L of MI, DCI, SI, or glucose. Mean values and standard deviations for three biological replicates are shown. a.u., arbitrary units.

In contrast to strain MB001(DE3)(pMKEx2-*eyfp*), the ΔIDH(pMKEx2-*eyfp*) strain was unable to grow on MI, SI, and DCI, confirming that this mutant is suitable to test the functionality of the different IDHs. Growth of the ΔIDH strain on MI comparable to that of the positive control was obtained by expressing either *iolG* or *oxiD* (Fig. 3). Expression of *oxiB* and *oxiE* also enabled growth on MI but at lower growth rates of 0.24 h^−1^ and 0.11 h^−1^, respectively. Expression of *iolW*, *oxiC*, and *idhA* did not restore growth on MI and showed the same profile as the negative control expressing *eyfp*.

Similar to the growth with MI, the growth of the ΔIDH strain with DCI was made possible by the expression of either *iolG* or *oxiD* (Fig. 3) and enabled the same growth rate (0.45 h^−1^) as that of the positive-control strain. In contrast to growth with MI, no growth on DCI was observed for the ΔIDH strain expressing *oxiB* or *oxiE*. Growth of the ΔIDH strain on SI was enabled only by expressing *oxiE* or *oxiB* (Fig. 3). Plasmid-based expression of *oxiE*, even at low induction levels, enabled faster growth (0.40 h^−1^) than that of the positive-control strain (0.26 h^−1^), indicating that native *oxiE* expression limited growth on SI. The expression of *oxiB* led to slower growth on SI (0.09 h^−1^) and a lower final backscatter after 48 h of cultivation.

The results of the growth experiments suggest that IolG and OxiD function as efficient MI and DCI dehydrogenases. OxiB and OxiE also possess MI dehydrogenase activity but apparently not DCI dehydrogenase activity. OxiE probably has a high SI dehydrogenase activity, whereas OxiB has a weak activity for SI.

### Kinetic properties of the enzymes IolG, OxiD, OxiE, and OxiB.

To confirm the conclusions derived from the growth experiments, we biochemically characterized those IDHs that enabled growth on the tested inositols, i.e., IolG, OxiD, OxiB, and OxiE. The enzymes were overproduced with a C-terminal Strep-tag II in C. glutamicum MB001(DE3) using the newly constructed pPREx6 vector. It enables the direct fusion of the target protein to a C-terminal Strep-tag II and strong inducible overexpression under the control of the T7 promoter. Enzymes were purified via StrepTactin Sepharose affinity chromatography followed by size exclusion chromatography. The purity of the proteins was confirmed by SDS-PAGE and Coomassie blue staining (Fig. S3).

The purified proteins were used for enzyme activity measurements via spectrophotometric assays measuring the decrease in absorbance of NADH at 340 nm with MI, SI, and DCI as the substrates. The results of these experiments agreed with the conclusions derived from the growth experiments and revealed clear differences in substrate preferences and activities (Table 1 and Fig. S4). IolG and OxiD both accept MI and DCI as the substrates with a preference for MI. OxiD showed a 2.5-times-higher turnover number for MI than IolG. Also, the *K_m_* of OxiD for MI was 3 times lower than that of IolG. OxiB showed activity for MI and SI but not for DCI. The specific activity of OxiB for MI was 4 times lower than that of IolG and 10 times lower than that of OxiD, corresponding to the slower growth of the ΔIDH(pMKEx2-OxiB) strain on MI (Fig. 3). The *K_m_* values of OxiB for MI and SI were similar and comparable to the *K_m_* values of IolG for MI. OxiE was the only IDH that showed activity for all three tested inositols with the highest activity and lowest *K_m_* for SI. The activity for DCI was more than 1,000-fold lower than that for MI and SI. This low activity was apparently not sufficient to enable the growth of the ΔIDH(pMKEx2-OxiE) strain on DCI.

**TABLE 1:** Role of the indicated IDHs for growth on MI, DCI, and SI and kinetic constants for oxidation of MI, DCI, and SI by purified IolG, OxiB, OxiD, and OxiE

Enzyme	Substrate	Growth^*a*^	*V*_max_ (μmol min^−1^ mg^−1^)	*K_m_* (mM)	*k*_cat_ (s^−1^)	*k*_cat_/*K_m_* (M^−1^ s^−1^)
IolG	*myo*-Inositol	+++	23.1 ± 3.2	60.9 ± 13.7	14.0 ± 1.9	235.1 ± 23.4
	d-*chiro*-Inositol	+++	14.3 ± 0. 9	61.93 ± 5.61	8.66 ± 0.54	140.39 ± 7.4
	*scyllo*-Inositol	−	ND^*b*^			

OxiD	*myo*-Inositol	+++	59.0 ± 2.3	19.6 ± 1.8	35.6 ± 1.4	1,831.8 ± 107.4
	d-*chiro*-Inositol	+++	25.5 ± 2.7	50.6 ± 8.7	15.4 ± 1.7	307.6 ± 20.0
	*scyllo*-Inositol	−	ND			

OxiB	*myo*-Inositol	++	5.8 ± 0.5	62.1 ± 12.5	4.2 ± 0.4	69.1 ± 7.3
	d-*chiro*-Inositol	−	ND			
	*scyllo*-Inositol	+	0.05 ± 0.01	28.8 ± 7.1	0.03 ± 0.00	1.03 ± 0.11

OxiE	*myo*-Inositol	+	3.1 ± 0.2	51.6 ± 3.6	3.9 ± 0.3	76.7 ± 5.2
	d-*chiro*-Inositol	−	0.005 ± 0.001	54.1 ± 8.0	0.003 ± 0.00	0.06 ± 0.00
	*scyllo*-Inositol	+++	13.4 ± 0.1	12.4 ± 0.8	8.5 ± 0.1	688.6 ± 41.5

a Growth of ΔIDH strain expressing the genes encoding the indicated IDHs, with +++ indicating very good growth and − indicating no growth.

b ND, no detectable activity.

### Analysis of the C. glutamicum IDHs by sequence alignments.

Explanations for the substrate specificity of different IDHs are scarce. Previous studies dealt with the structure elucidation of IDHs in complex with inositols to understand the interactions between the enzyme, the substrate, and the cofactor. For OxiD of C. glutamicum, a crystal structure (PDB ID 3EUW) with a resolution of 2.3 Å has been deposited in the Protein Data Bank (PDB) (44, 45) but without a bound cofactor or a substrate. The structure of IolG of B. subtilis complexed with NAD^+^ and MI enabled the identification of important residues for cofactor and substrate binding. Structure-based sequence alignments led to the definition of six conserved sequence motifs (46). Motifs I and II contain amino acid residues that are important for cofactor binding, whereas motifs III to VI contain residues responsible for substrate binding and the catalytic triad consisting of Lys97, Asp172, and His176 (BsIolG numbering).

**FIG 4:**
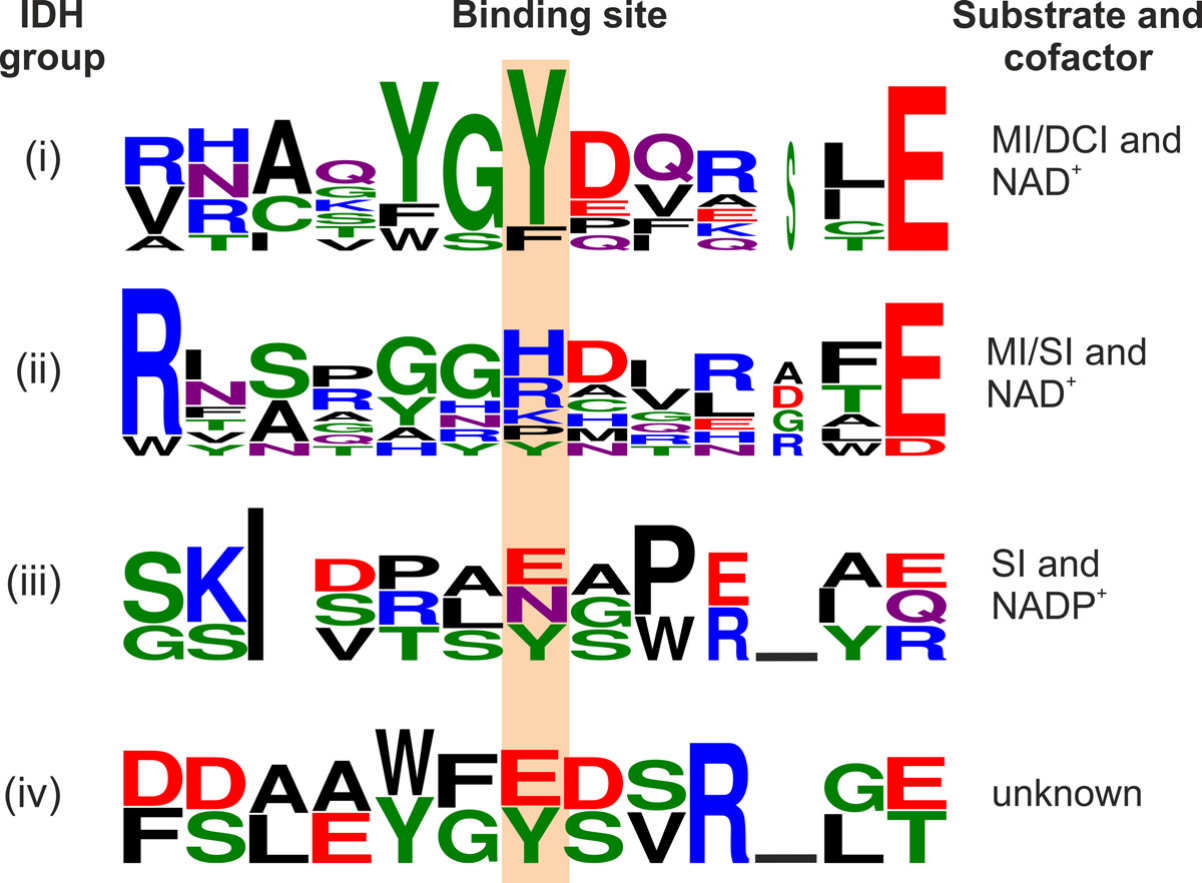
Consensus sequences for motif V of the four IDH subgroups with the substrate binding site highlighted. Letter height is proportional to the relative abundance of that residue at each position, and letter width is proportional to the fraction of valid symbols at that position. Letter color corresponds to the chemical properties of the amino acid (black, hydrophobic; red, acidic; blue, basic; green, polar; purple, carboxamides). The figure was generated using WebLogo 3 (WebLogo 3 - About [https://threeplusone.com]).

We compared the amino acid sequences of all seven annotated IDHs of C. glutamicum with the sequences of the biochemically characterized inositol dehydrogenases reported in the literature (Table S1) and sorted them into four groups (Fig. S5): (i) NAD^+^-dependent IDHs known to have activity for MI and DCI, (ii) NAD^+^-dependent IDHs known to have activity for MI and SI, (iii) NADP^+^-dependent IDHs catalyzing the reduction of 2KMI to SI, and (iv) IDHs with no activity for any tested inositol. In our comparison, we focused on the previously reported motifs to identify differences in functionally important residues within motifs I to VI. In group i, which includes CgIolG and CgOxiD, the sequences G_124_FM/NRRY/FD_130_ in motif III and Y_233_GY_235_ in motif V (BsIolG numbering) seem to be more conserved than in the other groups. F_125_M/N_126_R_127_ and Y235 were reported as substrate binding sites for BsIolG. In group ii, the sequence G_124_FM/NRRY/FD_130_ can also be found in some cases; however, the Y_233_GY_235_ sequence does not occur in any representative. In most cases, the second Tyr residue is replaced by a positively charged amino acid (H>R>K). As shown below, this residue is involved in substrate binding and, therefore, can serve as a marker to discriminate between IDHs specific for SI and DCI, although exceptions are possible (Fig. 4).

We previously identified IolW as an NADP^+^-dependent *scyllo*-IDH that catalyzes the reduction of 2KMI to SI (47) and therefore assigned it to group iii. At position 35, IolW contains an Ala residue, while most other IDHs contain an Asp or Glu residue. Asp or Glu residues at this position are conserved in NAD^+^-dependent IDHs, in which they form hydrogen bonds with the ribose moiety of NAD^+^. NADP^+^-dependent enzymes typically replace Asp or Glu with a small, neutral residue, as the negatively charged carboxylate of Asp or Glu would effectively repel the phosphate group in this position. Often, a basic residue follows the small neutral residue, like Arg36 in IolW, which can interact with the 2′-phosphate group of NADPH (48–50). Also, BsIolW and BsIolU, both of which have been characterized as NADPH-dependent KMI reductases, possess a Ser or Thr residue rather than Asp or Glu at position 35 (BsIolG numbering) (51). This difference between NAD^+^- and NADP^+^-dependent IDHs suggests that IolW is the only IDH of C. glutamicum favoring NADPH as a cofactor.

Among all analyzed IDHs, the motifs of CgOxiC differ the most from the published ones (Fig. S5). It is the only protein within the annotated IDHs of C. glutamicum that does not contain a complete GxGxxG consensus sequence in motif I. Additionally, instead of Asp179, a residue of the catalytic triad, OxiC contains an Ile residue. The lack of Asp179 suggests that OxiC is not active as an IDH, which is supported by the fact that expression of *oxiC* did not enable the growth of the ΔIDH strain on MI, DCI, or SI (Fig. 3). IdhA3 also differs at the corresponding position 172, as it contains a Glu residue instead of Asp, similar to the *myo*-IDH Gk1899, for which activity toward MI was reported previously (40). Despite being a conservative exchange, the difference in size might prevent IDH activity of IdhA3. As in the case of OxiC, the expression of *idhA3* did not allow growth of the ΔIDH strain on MI, DCI, or SI (Fig. 3). We constructed an IdhA3 variant in which we replaced Glu172 by Asp. However, the expression of *idhA3-*E172D also did not allow growth of C. glutamicum ΔIDH on MI, DCI, or SI (data not shown).

### Structural models of C. glutamicum IDHs and inositol docking.

Amino acid sequence comparisons of IDHs allow the prediction of cofactors and potential substrates when looking at highly conserved motifs. However, structural models and docking experiments are required to further understand inositol preferences and binding mechanisms. To this end, we generated structural models with their corresponding cofactor of the IDHs IolG, OxiB, OxiC, OxiD, OxiE, and IdhA3 (Table S2). All structures show an intermediate to good global model quality (Table S2) and good local model quality near the inositol binding sites (Fig. S6), with regions of lower quality located mainly in the loops and the central tetrameric interface.

The models served as input for docking experiments using AutoDock3 (52) in combination with DrugScore^2018^ (53) to probe the potential interaction between MI, SI, or DCI and the catalytic site of each IDH. To test if a docked solution likely adopts a favorable position in the catalytic site, we measured the distance *d* between the C-2 atom in MI, the C-1 or C-6 atom in DCI, or any C atom in SI and the C-4 atom of the nicotinamide group from the NAD^+^ cofactor. As the orientation of the reactive carbon atoms toward the cofactor and the distance between these atoms is crucial for the reaction to take place, we considered a binding pose valid only if *d *was ≤ 5 Å. For validation of the docking approach, we performed redocking experiments using the X-ray structures of Lactobacillus casei IDH1 (PDB ID 4MIO) and *L. casei* IDH2 (PDB ID 4N54) in complex with MI and SI, respectively. All dockings converged perfectly, and the poses show root mean square deviation (RMSD) values of <2.0 Å to the respective bound MI and SI in the X-ray structures (Fig. S7A and B). Furthermore, the docked solutions of the inositols showed *d* of <5 Å for both IDHs (Fig. S7C and D), even if the docked MI poses are slightly rotated in a counterclockwise manner.

**FIG 5:**
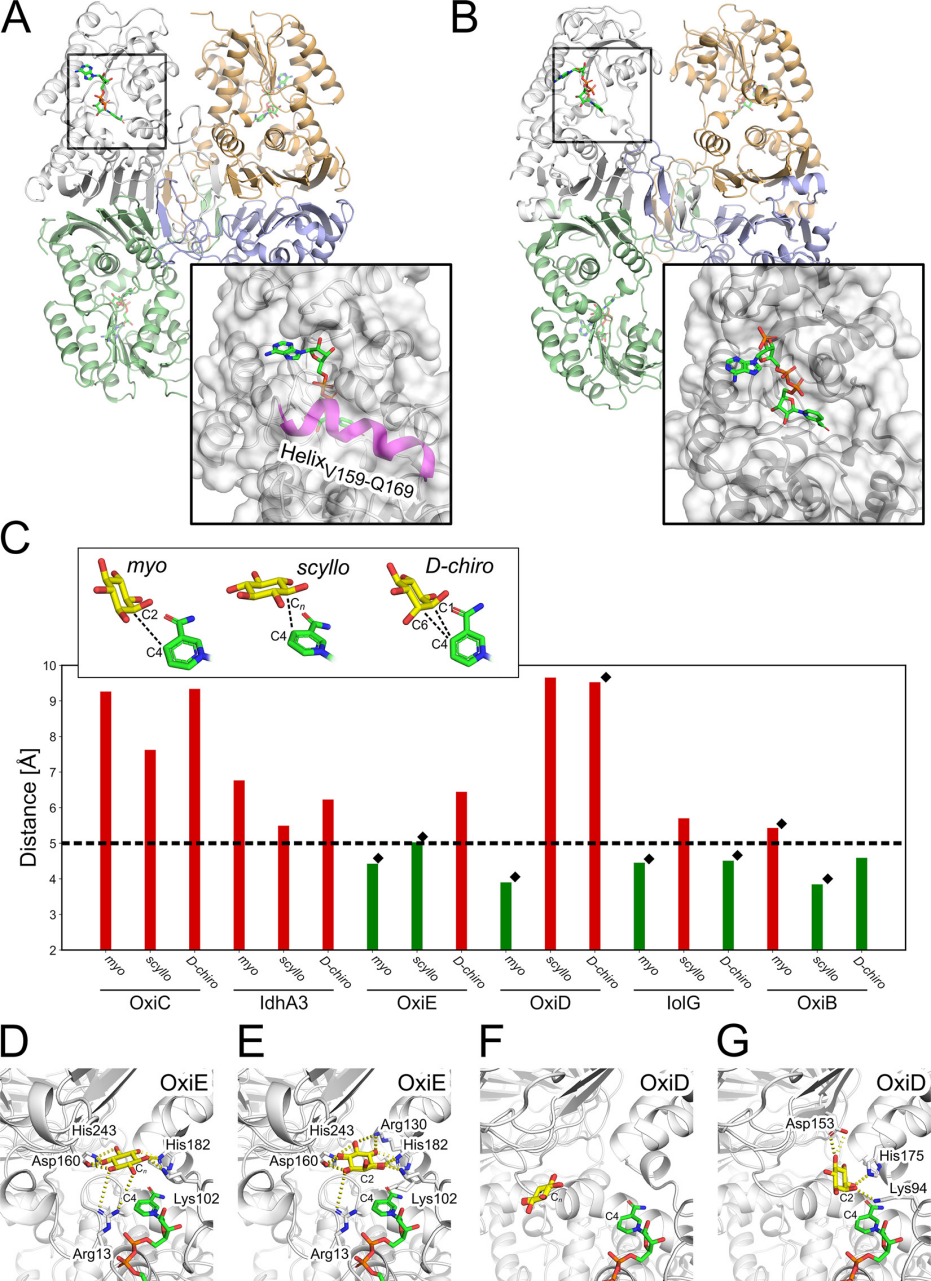
Docking results for *myo*-, *scyllo*-, and *d-chiro*-inositol into structural models of C. glutamicum IDHs. (A and B) Structural comparison between IdhA3 (A) and OxiC (B) shows an α-helix (magenta) blocking the entrance to the catalytic site of OxiC. Compared to active IDHs, the catalytic site in IdhA3 is more exposed. (C) Distances were measured for the docked inositol poses between the reactive carbon atom from each inositol and the C-4 atom of the cofactor nicotinamide group; black diamonds depict those inositols that result in growth. The horizontal dashed line indicates the threshold for considering a valid docking pose. The bar color depicts whether the distance is within the threshold (green) or outside (red). (D to G) Comparison of the docked solutions for MI and SI into OxiE and OxiD.

Of the systems investigated here, the two dehydrogenases, OxiC and IdhA3, served as negative controls as both show no activity for either MI or SI (47). The two IDHs differ markedly in the structure of the entrance region to the catalytic site. In OxiC, the presence of the α-helix V159 to Q169 narrows the catalytic site’s accessibility, thus hampering the interaction of the inositols and the enzyme (Fig. 5A). This helical element is missing in IolG, OxiD, OxiE, and IdhA3. The last shows an open catalytic site (Fig. 5B). In OxiB, an extended loop region is located at the same position as the helix in OxiC. However, the loop does not narrow the entrance to the catalytic site.

We obtained converged docking results for all combinations of these IDHs and the investigated inositols, indicating that the docking method finds a single most favorable binding pose for each inositol, except for the combinations of IdhA3 with MI and of OxiE with DCI, where two binding poses were found (Table S3). All docked solutions of the inositols in OxiC showed distances *d* >5 Å (Fig. 5C), since helix V159 to Q169 blocks the catalytic site. Despite the accessible catalytic site in IdhA3, we also observed no valid docking pose (*d* > 5 Å). This finding is remarkable as both IDHs showed no activity for MI or SI in previous experiments (47) and also could not recover growth of C. glutamicum ΔIDH on any tested inositol.

In the case of OxiE, valid docking poses were found for MI (*d *= 4.4 Å) and SI (*d *= 5.0 Å), which agrees with the activity data from experiments (Fig. 5C). Furthermore, the hydroxyl groups of docked inositol poses of MI and SI form interactions with the charged amino acids R13, K101, R130, D160, D178, H182, and H243 (Fig. 5D and E). Interestingly, we observed a slight incline of the docked MI compared to the SI orientation, which, though less pronounced, agrees with results reported previously for *L. casei* IDH1 (39). The docking with OxiE failed to generate valid docking poses for DCI, although purified OxiE showed very weak activity for DCI (Table 1), which was insufficient to enable growth on DCI.

For OxiD, we observed valid docking poses for MI (*d *= 3.9 Å) but not for SI (*d *= 9.7 Å), which agrees with the enzymatic activity data (Fig. 5C and Fig. S8). Here, the computed pose of MI interacts with the charged amino acids K94, D153, and H175 and the hydroxyl groups Y235 and Y280 (Fig. 5F and G). Compared to the orientation of the docked MI in OxiE, the incline is more pronounced in OxiD and similar to the orientation reported before for *L. casei* IDH1 (39). Surprisingly, for OxiD no valid docking pose was found for DCI (Fig. 5C), even though purified OxiD shows high activity for DCI as the substrate (Table 1).

For IolG, our docking results agree with the enzyme activity data (Fig. 5C). Here, MI and DCI produced valid docking poses (*d *= 4.5 Å for both inositols), whereas for SI no valid docking pose was found (*d *= 5.7 Å) (Fig. S8). Nevertheless, MI, SI, and DCI show the same interactions in our docking experiments with the amino acids H155, H176, S173, and Y235 (Fig. S9). The larger distance between SI and the cofactor is due to a wrong orientation of the inositol that shows an incline similar to that observed for MI. Finally, SI is located further away from the cofactor than MI and DCI.

In the case of OxiB, the predicted binding poses agree only for SI with the enzyme activity data (Fig. 5C). For SI, we observed a valid docking pose with a distance *d *of 3.8 Å (Fig. S8). The positions of all docked inositols are strongly overlapping, thus interacting with the same amino acids Y138, Y166, D193, H197, and N278 (Fig. S9). The larger distance between MI and the cofactor results from the misplaced C-2 atom of the inositol. Here, the C-2 atom points away from the cofactor and reveals a parallel orientation of the carbocyclic ring and the nicotinamide group of the cofactor. In the case of DCI, the C-1/C-6 atoms are oriented toward the cofactor and show a slight incline, suggesting an optimal interaction between DCI and the cofactor. Here, the docking result deviates from the enzyme activity data, as OxiB showed no activity with DCI as the substrate (Table 1). Overall, we were able to identify valid binding poses for MI, SI, and DCI in six IDHs in 15 out of 18 docking experiments.

## DISCUSSION

**FIG 6:**
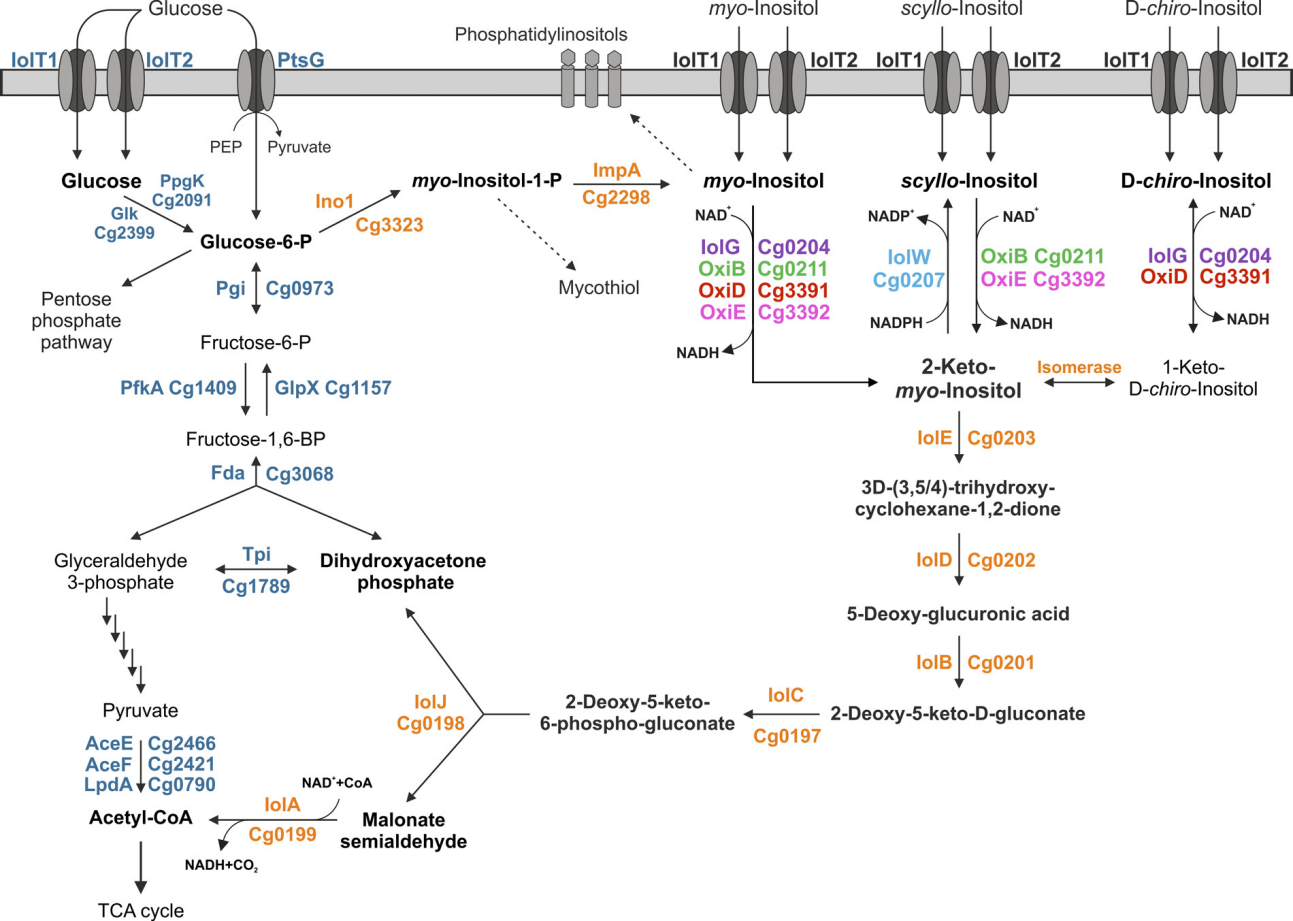
Schematic overview of *myo*-, *scyllo*-, and d*-chiro*-inositol catabolism in C. glutamicum. Reactions leading to cell constituents requiring l-*myo*-inositol-1-phosphate or *myo*-inositol for synthesis are indicated with dashed arrows. TCA, tricarboxylic acid.

The genome of C. glutamicum harbors seven genes that potentially encode IDHs. In this study, we characterized the physiological functions and biochemical properties of these IDHs and employed bioinformatics and molecular modeling to obtain more detailed information on their structural differences and substrate preferences. Our initial growth experiments with the strain MB001(DE3) derived from C. glutamicum ATCC 13032 revealed that it can grow not only with MI but also with SI and DCI as sole carbon and energy sources. In Fig. 6, we present an overview of our current knowledge of the inositol metabolism in C. glutamicum. We showed that both inositol transporters, IolT1 and IolT2, catalyze the uptake not only of MI but also of DCI and SI, which further underlines the fact that these transporters have a broad substrate specificity including not only inositols but also glucose, fructose, and xylose (54–57). According to the observed growth rates, IolT1 seems to have a higher activity for DCI and SI uptake than IolT2, while the two transporters are comparably effective with respect to MI uptake (35). Studies on the three inositol transporters of B. subtilis showed that they exhibit different preferences for different inositols (58).

By creating the ΔIDH strain of C. glutamicum, which lacks all seven known or putative IDH genes, we were able to test the role of each of the IDHs individually for their ability to enable growth on MI, SI, and DCI. The experiments showed that, besides IolG, also OxiD allows for fast growth on MI. OxiB and OxiE also enabled growth on MI but at a much lower rate. These results agree with the kinetic properties of the purified enzymes with MI as the substrate: IolG and OxiD showed *V*_max_ values about 5- to 10-fold-higher than those of OxiB and OxiE (Table 1). Growth on DCI was possible only with IolG and OxiD, and the kinetic properties confirmed a high activity of these enzymes with DCI as the substrate. OxiE showed superior growth and faster kinetics for SI than for MI, suggesting that this enzyme primarily functions as a *scyllo*-IDH. OxiB also allowed growth on SI but at a much lower rate. The kinetic properties of OxiE and OxiB for SI were in agreement with the growth data (Table 1). The observation that the IDHs possess activity for more than one inositol isomer has also been reported for IDHs of other bacteria. They are often classified either as *myo*-IDH with the highest activity for MI and lower activity for DCI or as *scyllo*-IDH with a preference for SI and lower activity for MI (21, 22, 39). OxiB is unusual in that it shows activity for SI and MI but has a strong preference for MI.

Expression of *iolW*, *oxiC*, and *idhA3* did not allow growth of the ΔIDH strain on MI, DCI, or SI, indicating that these proteins do not possess the required enzymatic activities. For IolW, this result was expected as our previous studies showed that this enzyme catalyzes the NADPH-dependent reduction of 2KMI to SI (47). In the case of OxiC, several reasons for the lack of enzymatic activity were identified. OxiC lacks the Asp179 residue, which is part of the catalytic triad, and contains an incomplete GxGxxG motif. Furthermore, the structural model shows that the substrate binding site of OxiC is blocked by an α-helix (Fig. 5A). Also, the docking experiments revealed no valid binding poses for the tested inositols. Therefore, all evidence argues against an enzymatic activity of OxiC as an IDH, and the function of this protein remains unknown. In the case of IdhA3, the Asp172 residue of the catalytic triad is replaced by a Glu residue and the exchange of the Glu residue with Asp did not enable growth on MI, SI, or DCI (data not shown). The structural model of IdhA3 shows that the catalytic site is more exposed than the binding pockets of active IDHs (Fig. 5B), and the docking experiments revealed no valid binding poses. As in the case of OxiC, the function of IdhA3 is currently unclear.

The structural models generated for the C. glutamicum IDHs in this study were used in blind docking experiments, which in 15 out of 18 cases were in good agreement with the experimental growth and kinetic data when the distance between the reactive carbon atom from each inositol and the C-4 atom of the cofactor’s nicotinamide group was evaluated as a criterion for activity. Only for the pairs OxiD/DCI and OxiB/MI, we obtained false-negative and, for OxiB/DCI, false-positive docking solutions, which might be due to treating the protein and the cofactor as rigid. Using computationally more demanding investigations including molecular dynamics simulations may overcome these limitations.

Amino acid sequence alignments and the predicted interactions of the inositols with the residues of the binding pocket suggested that motif V plays a role in the selectivity of IDHs. In group i, in which IDHs use MI and DCI as the substrates, a YGY_245_ motif (BsIolG numbering) with Y245 serving as a substrate binding site is highly conserved, whereas this motif is absent in group ii, in which IDHs use MI or SI as the substrate and in group iii, in which IDHs serve as 2KMI reductases. In group ii, Y245 is exchanged mainly for a positively charged residue (H>R>K) and the model proposes H243 as a substrate interaction site in OxiE (Fig. 5D and E). Therefore, this position can be used to estimate the substrate preferences of IDHs (Fig. 4).

As several inositols were reported to show pharmacological activities against, e.g., Alzheimer’s disease, polycystic ovarian syndrome, or type II diabetes (2), the biotechnological production of these sugar alcohols is of high interest and was shown, e.g., for SI production with B. subtilis (59) and C. glutamicum (47). These processes require the activity of IDHs for epimerization of MI to the desired inositol, and the knowledge of biochemical properties of the IDHs is a prerequisite for the design of appropriate synthesis pathways and chassis strains preventing, for example, the reoxidation of the target inositol. The structural models enable rational engineering of the IDHs to change the substrate or cofactor selectivity, which can provide new synthetic routes for the interconversion of inositol isomers. Besides MI, DCI, and SI, also other inositols were reported to have pharmacological activities (2). Our strategy for analyzing the properties of IDHs can be employed to identify novel IDHs suitable for production of rare inositols.

Our study revealed the functions of four of the seven putative IDHs present in C. glutamicum (IolG, OxiD, OxiB, and OxiE), not including the 2KMI reductase IolW reported previously (47). The functions of OxiC and IdhA3 remain unknown, and especially, OxiC is unlikely to be an active IDH. The overlapping substrate specificities of several of the four active NAD^+^-dependent IDHs might provide an advantage for scavenging inositols in the natural habitat. The oxidation product of MI and SI is 2KMI (or *scyllo*-inosose), which is subsequently converted by a 2KMI dehydratase (IolE) to 3D-(3,5/4)-trihydroxy-cyclohexane-1,2-dione (Fig. 6). The oxidation product of DCI, however, is 1-keto-d*-chiro*-inositol, which in B. subtilis is converted by the isomerase IolI to 2KMI (43). Current studies aim at identifying the C. glutamicum isomerase involved in growth on DCI.

## MATERIALS AND METHODS

### Bacterial strains, plasmids, and growth conditions.

All bacterial strains and plasmids used in this work are listed in Table 2. All cloning steps were performed with Escherichia coli DH5α as host. E. coli strains were cultivated at 37°C on LB agar plates or in lysogeny broth (LB) (60) with 50 μg/mL kanamycin. For growth characterization, C. glutamicum was cultivated in a BioLector microcultivation system (m2p-labs, Baesweiler, Germany). Single colonies were transferred in brain heart infusion (BHI) medium and cultivated for 8 h at 30°C as a first preculture. The second preculture containing defined CGXII medium (61) with 0.03 g/L protocatechuic acid and 2% (wt/vol) glucose was inoculated with 10% (vol/vol) of the first preculture and cultivated for 16 h at 30°C. Before inoculation of the main cultures, cells were washed once with CGXII medium without a carbon source. BioLector microcultivation was performed in 800 μL CGXII medium, which was supplemented with 1% (wt/vol) of the indicated carbon source in 48-well FlowerPlates (m2p-labs, Baesweiler, Germany) at 1,200 rpm at 30°C. Growth in this system was measured online as scattered light at 620 nm (62). For protein production, C. glutamicum was cultivated in 200 mL BHI medium supplemented with 2% (wt/vol) glucose in 2-L baffled shake flasks at 100 rpm and 30°C. When appropriate, 25 μg/mL kanamycin was added to the medium. Gene expression was induced via the addition of isopropyl-β-d-thiogalactoside (IPTG) at the indicated concentrations. Bacterial growth was followed by measuring the optical density at 600 nm (OD_600_).

**TABLE 2:** Bacterial strains and plasmids used in this study

Strain or plasmid	Relevant characteristics	Reference or source
Strains		
E. coli DH5α	F^−^ ϕ80d*lac*Δ(*lacZ*)M15 Δ(*lacZYA-argF*)*U169 endA1 recA1 hsdR17* (r_K_^−^ m_K_^+^) *deoR thi-1 phoA supE44* λ^−^ *gyrA96 relA1*; strain used for cloning procedures	65
C. glutamicum		
MB001(DE3)	Derivative of the prophage-free strain MB001 with a chromosomally encoded E. coli *lacI* gene under control of its native promoter followed by the T7 RNA polymerase gene under control of the *lacUV5* promoter	78
MB001(DE3)ΔIDH	MB001(DE3) derivative with deletion of the genes *oxiC*-cg3390-*oxiD*-*oxiE* (cg3389–cg3392), *iolG* (cg0204), *iolW* (cg0207), *idhA3* (cg2313), and *oxiB* (cg0211)	This work
ATCC 13032 Δ*iolT1*	Derivative of the wild-type ATCC 13032 in which the inositol transporter *iolT1* was deleted	35
ATCC 13032 Δ*iolT2*	Derivative of the wild-type ATCC 13032 in which the inositol transporter *iolT2* was deleted	35
ATCC 13032 Δ*iolT1*Δ*iolT2*	Derivative of the wild-type ATCC 13032 in which the inositol transporters *iolT1* and *iolT2* were deleted	35
Plasmids		
pK19mobsacB	Kan^r^; plasmid for allelic exchange in C. glutamicum; pK18 *ori*V*_E.c_*_._ *sacB lacZ*α	79
pK19mobsacBΔ*iol2*	Kan^r^; plasmid for deletion of the genes cg3389–cg3392 containing two 1-kb PCR products which cover the upstream flanking region of *oxiC* (cg3389) and the downstream flanking region of *oxiE* (cg3392)	47
pK19mobsacBΔ*iolG*	Kan^r^; plasmid for deletion of *iolG* (cg0204)	80
pK19mobsacBΔ*iolW*	Kan^r^; plasmid for deletion of *iolW* (cg0207)	57
pK19mobsacBΔ*oxiB*	Kan^r^; plasmid for deletion of *oxiB* (cg0211)	This work
pK19mobsacBΔ*idhA3*	Kan^r^; plasmid for deletion of *idhA3* (cg2313)	This work
pMKEx2	Kan^r^; *E. coli*-*C. glutamicum* shuttle vector (*lacI* P_T7_ *lacO1* pHM1519 *ori_Cg_*; pACYC177 *ori_Ec_*) for expression of target genes under control of the T7 promoter	78
pMKEx2-*eyfp*	Kan^r^; pMKEx2 derivative containing the *eyfp* gene under control of P_T7_	78
pMKEx2-IolG	Kan^r^; pMKEx2 derivative containing the *iolG* gene under control of P_T7_	This work
pMKEx2-IolW	Kan^r^; pMKEx2 derivative containing the *iolW* gene under control of P_T7_	This work
pMKEx2-OxiB	Kan^r^; pMKEx2 derivative containing the *oxiB* gene under control of P_T7_	This work
pMKEx2-IdhA3	Kan^r^; pMKEx2 derivative containing the *idhA3* gene under control of P_T7_	47
pMKEx2-OxiC	Kan^r^; pMKEx2 derivative containing the *oxiC* gene under control of P_T7_	47
pMKEx2-OxiD	Kan^r^; pMKEx2 derivative containing the *oxiD* gene under control of P_T7_	47
pMKEx2-OxiE	Kan^r^; pMKEx2 derivative containing the *oxiE* gene under control of P_T7_	47
pPREx2	Kan^r^; *E. coli*-*C. glutamicum* shuttle vector (P*_tac_ lacI*^q^ pBL1 *ori_Cg_*; ColE1 *ori_Ec_* with a Strep-tag II-encoding sequence	68
pPREx6	Kan^r^; pPREx2 derivative with P*_tac_* exchanged for P_T7_ promoter	This work
pPREx6-IolG	Kan^r^; pPREx6 derivative containing the *iolG* gene under control of P_T7_ and fused to Strep-tag II sequence	This work
pPREx6-OxiB	Kan^r^; pPREx6 derivative containing the *oxiB* gene under control of P_T7_ and fused to Strep-tag II sequence	This work
pPREx6-OxiD	Kan^r^; pPREx6 derivative containing the *oxiD* gene under control of P_T7_ and fused to Strep-tag II sequence	This work
pPREx6-OxiE	Kan^r^; pPREx6 derivative containing the *oxiE* gene under control of P_T7_ and fused to Strep-tag II sequence	This work

### Recombinant DNA work and construction of deletion mutants.

Plasmids and oligonucleotides used in this study are listed in Table 2 and in Table S4 in the supplemental material, respectively. PCRs, DNA restrictions, and plasmid constructions were performed according to established protocols (63, 64). DNA sequencing and oligonucleotide synthesis were performed by Eurofins Genomics (Ebersberg, Germany). Chemically competent E. coli cells were transformed according to an established protocol (65). C. glutamicum was transformed via electroporation as described previously (66). The deletion mutant C. glutamicum MB001(DE3)ΔIDH was constructed via consecutive double homologous recombination as described previously (67) using the plasmids pK19mobsacBΔiol2, pK19mobsacBΔiolG, pK19mobsacBΔiolW, pK19mobsacBΔidhA3, and pK19mobsacBΔoxiB. The chromosomal deletions were confirmed via colony PCR using oligonucleotides annealing outside the deleted region.

For the construction of the pMKEx2-based expression plasmids, the corresponding target genes were cloned downstream of the C. glutamicum consensus ribosome binding site (RBS) via Gibson assembly. For protein overproduction and purification, the inositol dehydrogenase genes were cloned into the newly constructed pPREx6 plasmid, which is a derivative of pPREx2 (68) in which the promoter P*_tac_* was replaced by the T7 promoter. For promoter exchange, the plasmid backbone was amplified using oligonucleotides P027 and P028, and the T7 promoter was amplified from pMKEx2 with oligonucleotides P029 and P030. DNA fragments were joined via Gibson assembly, yielding pPREx6.

### Protein overproduction and purification.

C. glutamicum MB001(DE3) was transformed with pPREx6-based expression plasmids for inositol dehydrogenase production and cultivated in 200 mL BHI medium supplemented with 20 g/L glucose. Gene overexpression was induced with 250 μM IPTG after 3 h, and cells were harvested after 24 h of cultivation via centrifugation at 5,000 × *g* for 20 min at 4°C. Cell pellets were washed and resuspended in 4 mL lysis buffer (100 mM KPO_4_, pH 7.5, 150 mM NaCl, 1 mM MgSO_4_) per g (wet weight) of cells and lysed by five passages through a French press at 124 MPa. The resulting cell extract was first centrifuged at 5,000 × *g* and 4°C for 20 min, and the supernatant was then subjected to ultracentrifugation at 45,000 × *g* and 4°C for 1 h. The resulting supernatant was incubated with avidin (25 μg/mg protein) for 30 min on ice before performing purification on an Äkta pure protein purification system (Cytiva) via StrepTactin Sepharose affinity chromatography and subsequent size exclusion chromatography.

A StrepTrap HP 1-mL column was equilibrated with binding buffer (100 mM KPO_4_, pH 7.5, 150 mM NaCl, 1 mM MgSO_4_) before loading the protein extract. The column was washed with 10 column volumes (CV) of binding buffer, and the remaining proteins were then eluted in six 0.5-mL fractions with elution buffer I (100 mM KPO_4_, pH 7.5, 150 mM NaCl, 1 mM MgSO_4_, 2.5 mM dethiobiotin). The protein-containing elution fractions were combined and concentrated by using a 10-kDa Amicon filter and centrifuging at 3,500 × *g* and 4°C to a final volume of 500 μL. The concentrated protein was then applied to a Superdex 200 Increase size exclusion chromatography column that had been equilibrated with 2 CV of elution buffer II (100 mM KPO_4_, pH 7.5, 1 mM MgSO_4_). Protein was eluted with 1.5 CV of elution buffer II and collected in 2-mL fractions. The purity and apparent molecular mass of the proteins after both purification steps were determined by 12% (wt/vol) SDS-PAGE according to standard procedures (64). Protein concentrations were determined using the Bradford assay (69).

### Inositol dehydrogenase activity assays.

Inositol dehydrogenase activity was determined as described before with some adjustments (68). Measurements were performed in a 600-μL reaction volume using 1-mL cuvettes containing 0.25 to 600 μg purified enzyme in elution buffer II at 30°C. A reaction mixture without the substrate was used as a blank, and the reaction was initiated by the addition of the substrate. Kinetic assays were performed with various concentrations of MI, DCI, and SI (0.5 to 50 mM) at a constant concentration of 5 mM NAD^+^. Kinetic constants were determined via a nonlinear regression fit based on the Michaelis-Menten equation with the GraphPad Prism software.

### Structural bioinformatics methods.

Homology models of the IDHs were generated with the protein structure homology modeling server of SWISS-MODEL (70, 71). The template search against the SWISS-MODEL template library (SMTL; last update 2 October 2021, last included PDB release 2 May 2021) was performed with BLAST (72) and HHblits (73): Initially, the target sequence was searched with BLAST against the primary amino acid sequences contained in the SMTL. A total of 23 (OxiC), 79 (IdhA3), 42 (OxiE), 61 (OxiD), 28 (IolG), and 19 (OxiB) templates were found. An initial HHblits profile was built using the procedure outlined in reference 73, followed by one iteration of HHblits against Uniclust30 (74). Next, the obtained profile was searched against all profiles of the SMTL. A total of 1,846 (OxiC), 3,608 (IdhA3), 2,552 (OxiE), 3,393 (OxiD), 3,946 (IolG), and 2,505 (OxiB) templates were found. Based on the found template structures, we chose the ones that included a bound cofactor and showed the highest sequence identity (Table S2). Models are built based on the target-template alignment using ProMod3 (75). Coordinates conserved between the target and the template are copied from the template to the model. Insertions and deletions are remodeled using a fragment library. Side chains are then rebuilt. Finally, the resulting model’s geometry is regularized using a force field. The global and per-residue model quality was assessed using the QMEANDisCo scoring function (76) (Fig. S6). The cofactors’ position was determined from the template structures and carried over to the structural models using PyMOL Molecular Graphics System, version 2.3.0 (Schrödinger, LLC, New York, NY) (77).

For the molecular docking, the three-dimensional (3D) structures of the substrates MI, SI, and DCI were generated based on their corresponding SMILES codes using RDKit: Open-source Chemoinformatics (https://doi.org/10.5281/zenodo.3732262). The substrates were subsequently docked into the catalytic sites of the respective IDH utilizing a combination of AutoDock3 (52) as a docking engine and DrugScore^2018^ (53) as an objective function. Docking grids were generated with DrugScore^2018^ using converged pair-potentials for all atom pairs. The position and dimension of the grids were calculated using the positions of inositols in crystal structures as reference points. Accounting for a margin of 8 Å in every direction, the final docking grid shows box dimensions of approximately 23 Å by 23 Å by 20 Å and is centered in the pocket of the IDHs (Fig. S7A and B). Following an established procedure (53), the docking protocol considered 100 independent runs for each ligand using an initial population size of 100 individuals, a maximum number of 27.0 × 10^3^ generations, a maximum number of 5.0 × 10^6^ energy evaluations, a mutation rate of 0.02, a crossover rate of 0.8, and an elitism value of 1. The Lamarckian genetic algorithm was chosen for sampling in all approaches. The distance between the reactive carbon of the docked substrates and the cofactor was measured using the PyMOL Molecular Graphics System.

### Data availability.

The strains and plasmids used in this work will be made available by the corresponding author (M.B.) upon request.
