# Supplementary material for: Physiological, Biochemical, and Structural Bioinformatic Analysis of the Multiple Inositol Dehydrogenases from Corynebacterium glutamicum
Source: Microbiol Spectr. 2022 Sep 12;10(5):e01950-22. doi: 10.1128/spectrum.01950-22 (PMC9603128; doi:10.1128/spectrum.01950-22)
Supplement: Supplemental file 1 — Tables S1 to S4; Fig. S1 to S9. Download spectrum.01950-22-s0001.pdf, PDF file, 4.0 MB [file spectrum.01950-22-s0001.pdf]

SUPPORTING INFORMATION

Physiological, biochemical, and structural bioinformatic analysis of the multiple inositol dehydrogenases from *Corynebacterium glutamicum*

Paul Ramp[1,2], Christopher Pfleger[3], Jonas Dittrich[2,3], Christina Mack[1], Holger Gohlke[2,3,4,#], and Michael Bott[1,2,#]

[1]IBG-1: Biotechnology, Institute of Bio- and Geosciences, Forschungszentrum Jülich, Jülich, Germany
[2]The Bioeconomy Science Center (BioSC), Forschungszentrum Jülich, Jülich, Germany
[3]Institut für Pharmazeutische und Medizinische Chemie, Heinrich-Heine-Universität Düsseldorf, 40225 Düsseldorf, Germany
[4]John von Neumann Institute for Computing (NIC), Jülich Supercomputing Centre (JSC), Institute of Biological Information Processing (IBI-7: Structural Biochemistry) & Institute of Bio- and Geosciences (IBG-4: Bioinformatics), Forschungszentrum Jülich GmbH, 52425 Jülich, Germany

[#]Corresponding authors: m.bott@fz-juelich.de and h.gohlke@fz-juelich.de

Running title: Inositol dehydrogenases of *Corynebacterium glutamicum*

Table S1. Identifiers and references for the characterized IDHs aligned in Fig. S5.

Name	Uniprot number	Organism	Reference
BsIolG	P26935	*B. subtilis*	(1)
BsIolW	O32223	*B. subtilis*	(2)
BsIolX	P40332	*B. subtilis*	(2)
BsIolU	O05265	*B. subtilis*	(3)
Gk1897	Q5KYQ4	*Geobacillus kaustophilus* strain HTA426	(4)
Gk1898	Q5KYQ3	*Geobacillus kaustophilus* strain HTA426	(4)
Gk1899	Q5KYQ2	*Geobacillus kaustophilus* strain HTA426	(4)
LcIDH1	E1U887	*Lactobacillus casei* strain Bl23	(5)
LcIDH2	E1U888	*Lactobacillus casei* strain Bl23	(5)
TmIolG	Q9WYP5	*Thermotoga maritima*	(6)
SMc01163	Q92SL5	*Rhizobium meliloti* strain 1021 (*Sinorhizobium meliloti* strain 1021)	(7)
SmIdhA	O68965	*Rhizobium meliloti* strain 1021 (*Sinorhizobium meliloti* strain 1021)	(8)
PllgdA	K7ZP76	*Paracoccus laeviglucosivorans*	(9)

Table S2. Used templates for generating structural models of the IDHs and global assessment of the model quality.

**IDH**	**Template PDB**	**Sequence identity [%]**	**Resolution [Å]**	**QMEANDisCo Global (10)**
IdhA3	4l8v	33.99	2.1	0.52
OxiC	3cea	23.05	2.4	0.55
OxiB	5ya8	33.33	2.3	0.67
OxiD	4mio	25.47	1.5	0.62
OxiE	4n54	35.88	2.1	0.65
IolG	4l8v	38.44	2.1	0.75

Table S3. Convergence of docking results[1]

IDH	*myo*-Inositol	*scyllo*-Inositol	D-*chiro*-Inositol
IdhA3	Cl 1: 98% (-6.31 kcal mol^{-1})<br>Cl 2: 2% (-6.28 kcal mol^{-1})	Cl 1: 100% (-6.23 kcal mol^{-1})	Cl 1: 100% (-6.40 kcal mol^{-1})
OxiC	Cl 1: 100% (-6.97 kcal mol^{-1})	Cl 1: 100% (-6.85 kcal mol^{-1})	Cl 1: 100% (-6.97 kcal mol^{-1})
OxiB	Cl 1: 100% (-7.11 kcal mol^{-1})	Cl 1: 100% (-7.02 kcal mol^{-1})	Cl 1: 100% (-6.96 kcal mol^{-1})
OxiD	Cl 1: 100% (-6.71 kcal mol^{-1})	Cl 1: 100% (-6.51 kcal mol^{-1})	Cl 1: 100% (-6.51 kcal $mol^{-1)}$
OxiE	Cl 1: 100% (-7.32 kcal mol^{-1})	Cl 1: 100% (-7.37 kcal mol^{-1})	Cl 1: 65% (-6.98 kcal mol^{-1})<br>Cl 2: 35% (-6.93 kcal mol^{-1})
IolG	Cl 1: 100% (-7.24 kcal mol^{-1})	Cl 1: 100% (-7.23 kcal mol^{-1})	Cl 1: 100% (-7.18 kcal mol^{-1})

[1] RMSD-based clustering of docked inositols; percentage convergence of docked inositol poses; average energies shown in parentheses.

Table S4. Oligonucleotides used in this study

Oligonucleotide name	Oligonucleotide sequence (5'→ 3')
pK19mobsacB plasmids	
P001_ΔoxiB_1	GCATGCCTGCAGGTCGACTCTAGAGTTGTCGTCGATGGTGGTG
P002_ΔoxiB_2	AAGTTCTTGCTGAGTCAC
P003_ΔoxiB_3	GGTCATAGTGACTCAGCAAGAACTTATCCCTGCAAACAACTAG
P004_ΔoxiB_4	CGTTGTAAAACGACGGCCAGTGAATTTCCTTGGTCACCAGATC
P005_Δcg2313_1	GAGGATCCCCGGGTACCGAGCTCGCCTCAAGCGGAACCTGAAG
P006_Δcg2313_2	CTTGCTGAAAGCATCGAGG
P007_Δcg2313_3	GTTAAACCTCGATGCTTTCAGCAAGGAGGGCAAGTTTGACTGAC
P008_Δcg2313_4	CGTTGTAAAACGACGGCCAGTGAATTATGGTGGTCAAGCCGATG
Recombination analysis	
P009_ΔiolG_1	CGACGTTGCTGGTCTTGCTTCCAAG
P010_ΔiolG_2	GGTTAGTGATGTAGCGCAGGCCGTG
P011_ΔiolW_1	AAGTGCTGCGGTGGTATGCGGTTTT
P012_ΔiolW_2	CTTGCGCGGGCGAGGAACTAACTCG
P013_ΔoxiB_1	ATGCATGATCTCCGGGTG
P014_ΔoxiB_2	CGGATATGACAACACTCC
P015_Δcg2313_1	GGAATGGGCTGCGTTG
P016_Δcg2313_2	GCAGAAGTTTCGGTGTG
P017_Δiol2_1	ACACCATCCGGGACAC
P018_Δiol2_2	ACTGCAATGCTGGCCTG
pMKEx2 plasmids	
P019_iolG_1	TTAACTTTAAGAAGGAGATATACCATGAGCAAGAGCCTTCGC
P020_iolG_2	CTTTCAGAAGTGGGTTTCTCCTTAAGCGTAGAAATC
P021_iolW_1	GATTTCTACGCTTAAGGAGAAACCCACTTCTGAAAG
P022_iolW_2	TGGCACCAGAGCGAGCTCTGCGGCCTTAGCTCAACTCAATGGTG
P023_oxiB_1	TTTAACTTTAAGAAGGAGATATACCATGACTCAGCAAGAACTTC
P024_oxiB_2	GCACCAGAGCGAGCTCTGCGGCCGCCTAGTTGTTTGCAGGGATC
P025_idhA3_E172D_1	GGACCATCTTCCTGGATACCCTCATCCACGATTTC
P026_idhA3_E172D_2	GAAATCGTGGATGAGGGTATCCAGGAAGATGGTCC
pPREx6 plasmids	
P027_pPREx2_1	GAAGGAGATATACATATGACCTGAGCTAGC
P028_pPREx2_2	GCCAGAACCGTTATGATG
P029_T7_ 1	CATCATAACGGTTCTGGCATGCGTCCGGCGTAGAG
P030_T7 _2	CTAGCTCAGGTCATATGTATATCTCCTTCTTAAAGTTAAACAAAATTATTTC
P031_x6_IolG_1	GCCTGCAGAAGGAGATATACAATGAGCAAGAGCCTTCGC
P032_x6_IolG_2	TTACTTCTCGAACTGTGGGTGGGACCAGCTAGCAGCGTAGAAATCTGGGCGAGG
P033_x6_OxiB_1	TTAACTTTAAGAAGGAGATATACATATGACTCAGCAAGAACTTC
P034_x6_OxiB_2	CGAACTGTGGGTGGGACCAGCTAGCCTAGTTGTTTGCAGGGATC
P035_x6_OxiD_1	TTTAAGAAGGAGATATACATATGACTCTTCGTATCGCC
P036_x6_OxiD_2	TGTGGGTGGGACCAGCTAGCCTAAACGTTGGCAGGGTTGAG
P037_x6_OxiE_1	TTTAAGAAGGAGATATACATATGAAAAACATCACCATCGG
P038_x6_OxiE_2	TGTGGGTGGGACCAGCTAGCTTAAGCAGATGGAACCAGCG

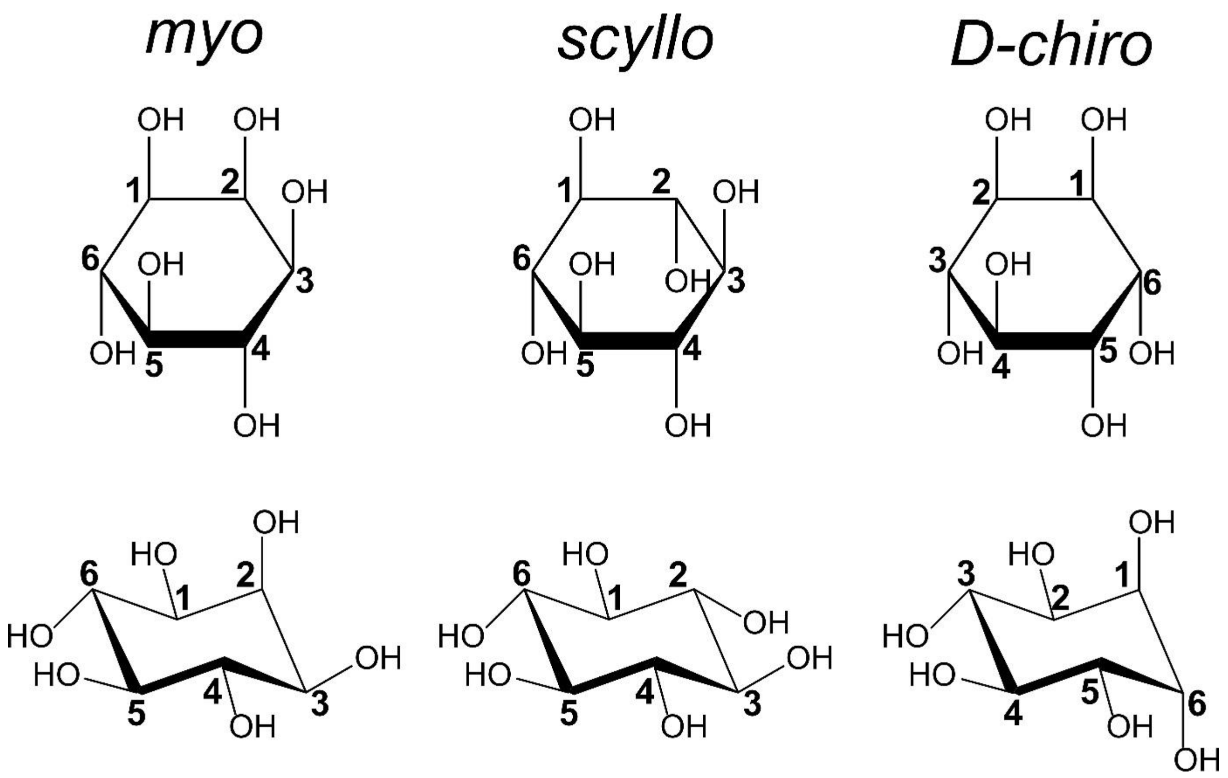

Fig. S1. Haworth projection and chair form of MI, SI, and DCI according to (11).

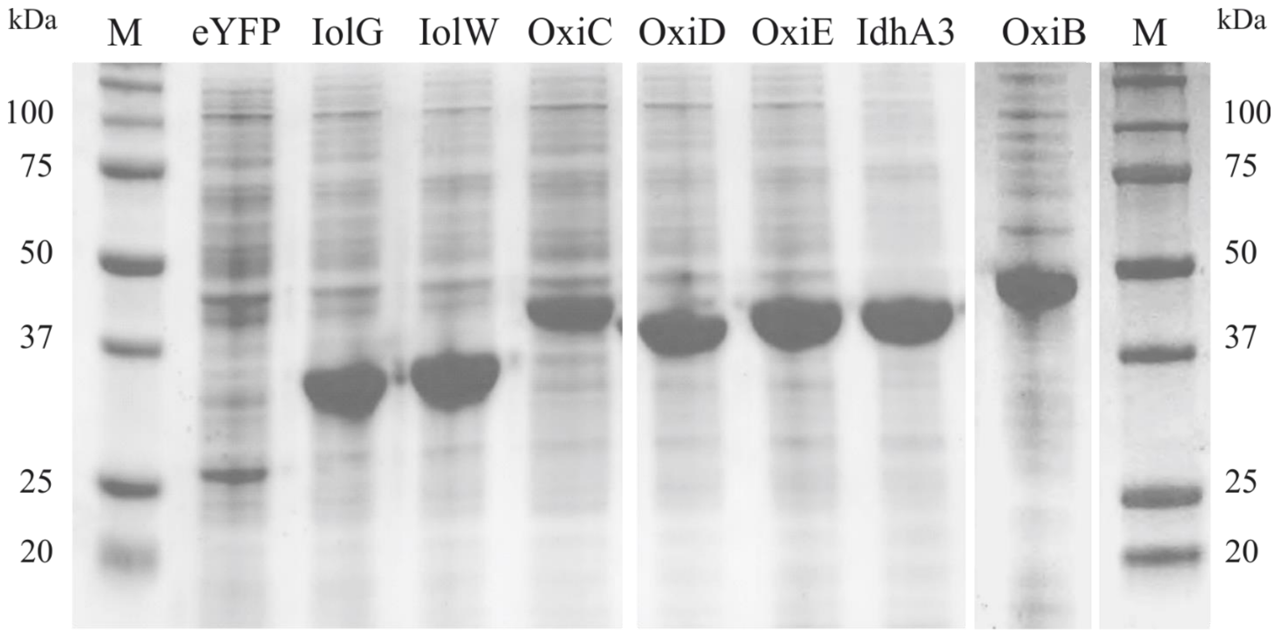

Fig. S2. SDS-PAGE of supernatant fractions of lysed *C. glutamicum* ΔIDH cells overproducing the indicated IDHs using pMKEx2-based expression plasmids. The gels were stained with Coomassie blue. M, protein standards; eYFP, control strain overproducing the eYFP protein (27 kDa).

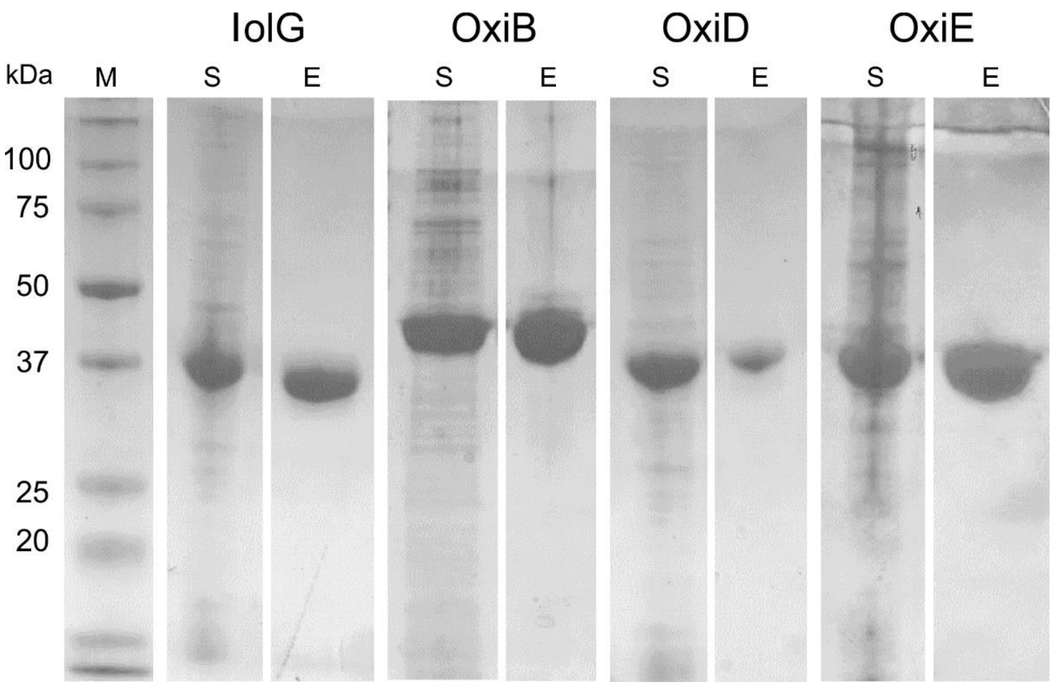

Fig. S3. Coomassie-stained SDS-polyacrylamide gels of purified IolG, OxiB, OxiD and OxiE. Shown are supernatant fractions of lysed *C. glutamicum* cells overexpressing the mentioned IDH (S) and final elution fractions after purification by affinity and size-exclusion chromatography.

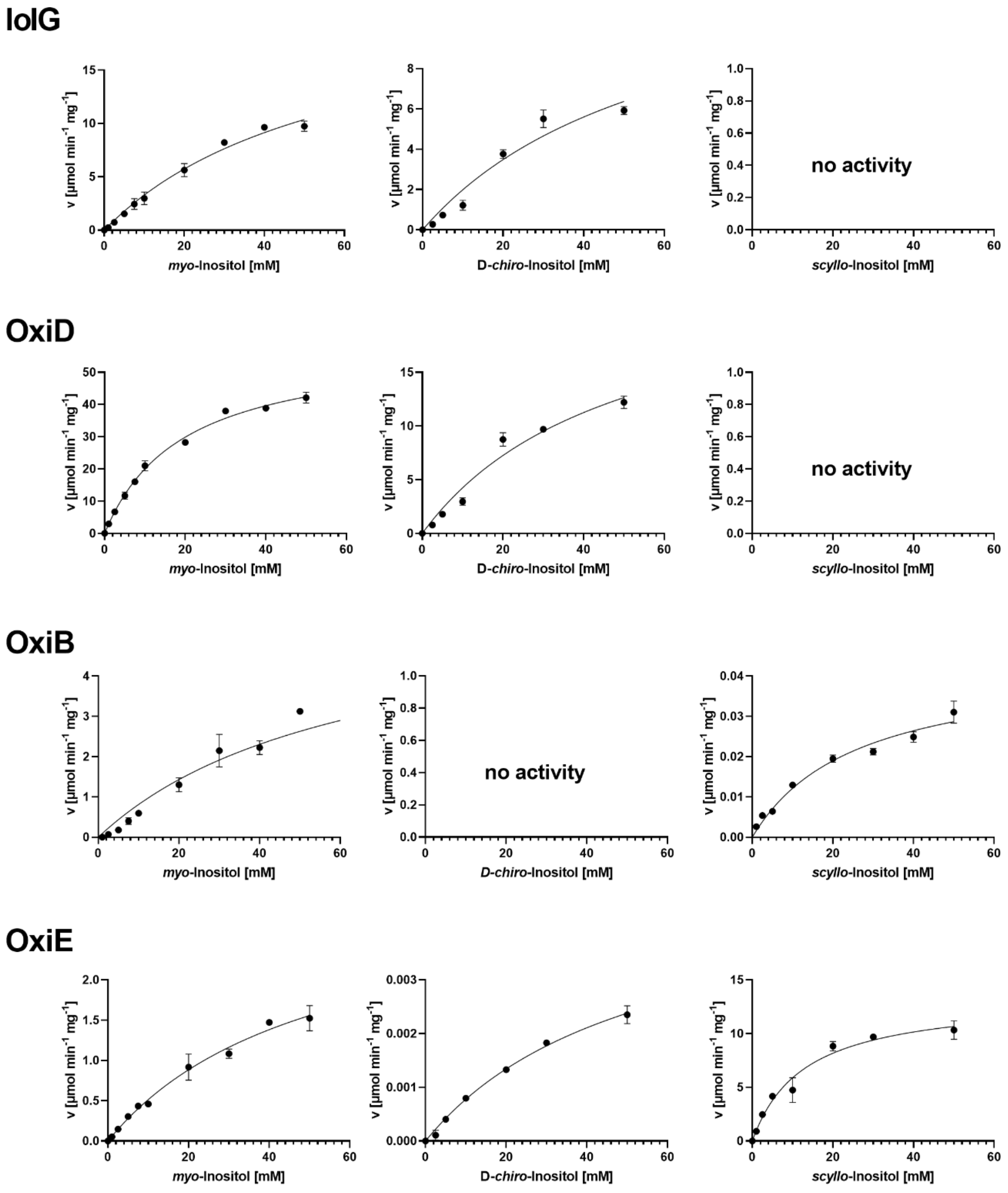

Fig. S4. Michaelis-Menten plots for the four indicated IDHs and the three different substrates MI, DCI, and SI. Kinetic constants were determined via a non-linear regression fit based on the Michaelis-Menten equation performed with the GraphPad Prism software.

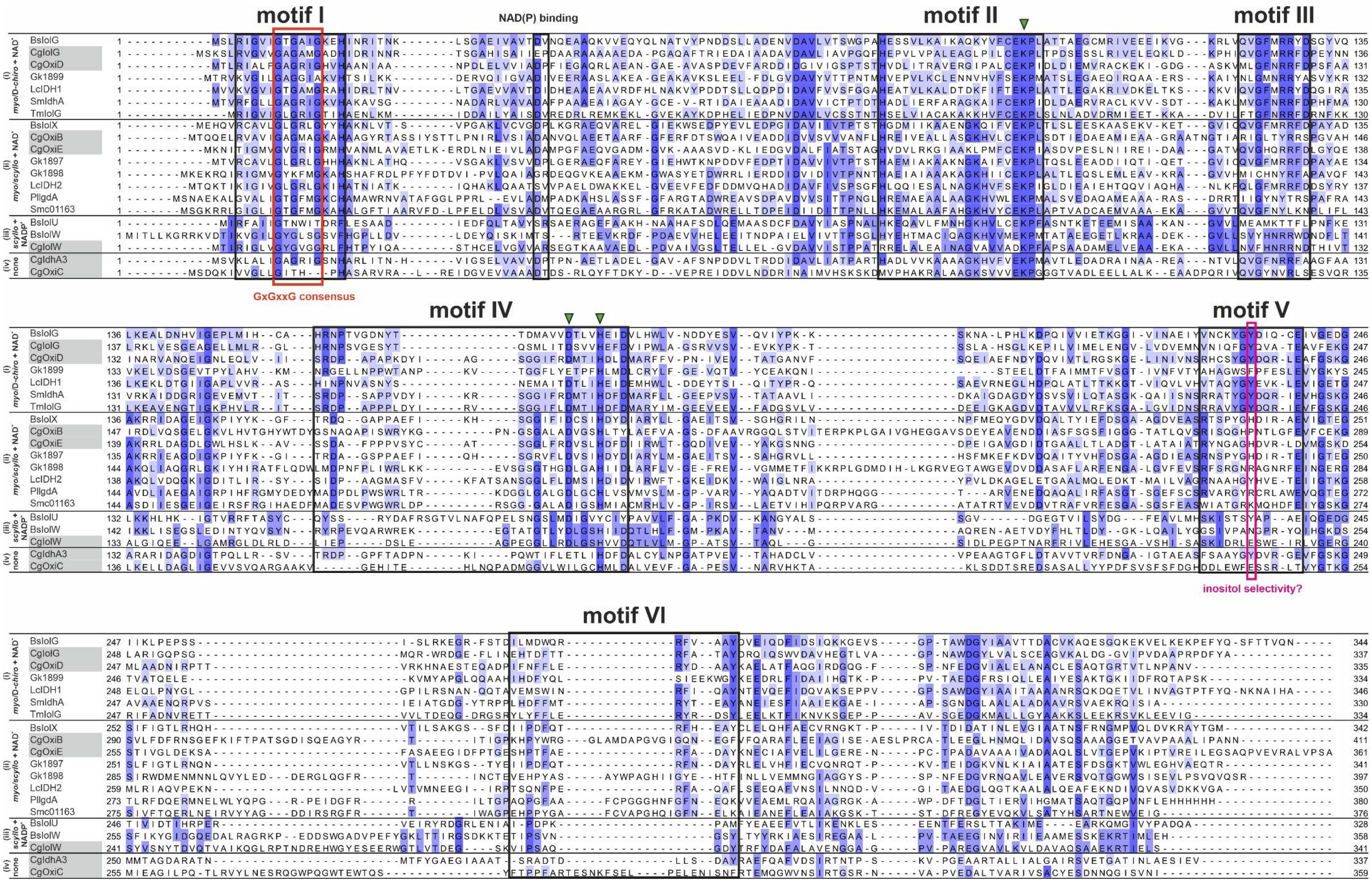
motif I
NAD(P) binding
motif II
motif III
GxGxxG consensus
motif IV
motif V
inositol selectivity?
motif VI
(i) myo/D-chiro + NAD+
(ii) myo/scyllo + NAD+
(iii) scyllo + NADP+
(iv) none

Fig. S5. Amino acid sequence alignment of the seven known or putative inositol dehydrogenases of *C. glutamicum* with characterized IDHs from other bacteria using Clustal Omega. Amino acid conservation for each subgroup is highlighted in blue color with intensity representing conservation rate. Highlighted are important residues and motifs for cofactor binding (motifs I and II with GxGxxG consensus sequence and residues 45-46) and for substrate binding and catalysis (motifs III-VI). The residues of the catalytic triade are highlighted by green arrows. The residue highlighted with a pink frame was identified to enable estimations of the substrate preferences of the IDHs (see text). The identifiers and references for the sequences from characterized IDHs of other bacteria are shown in Table S1.

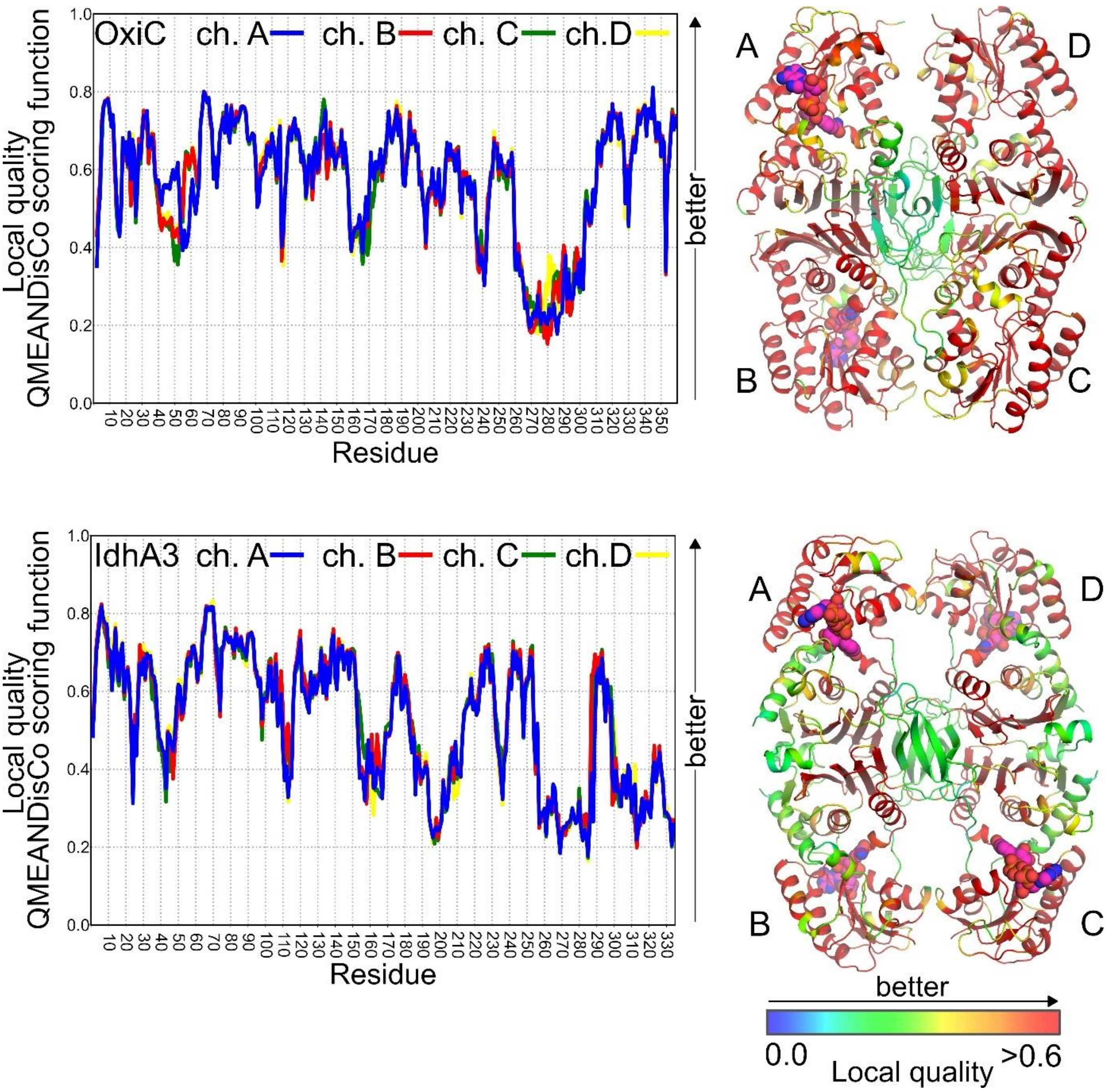

Continued next page

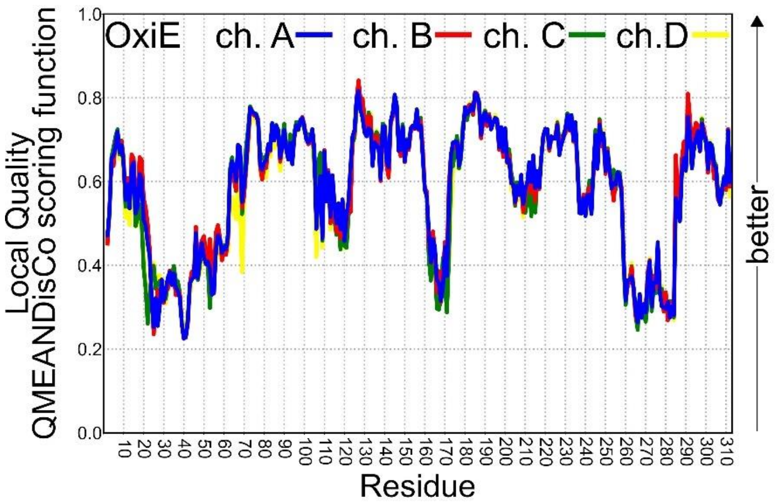

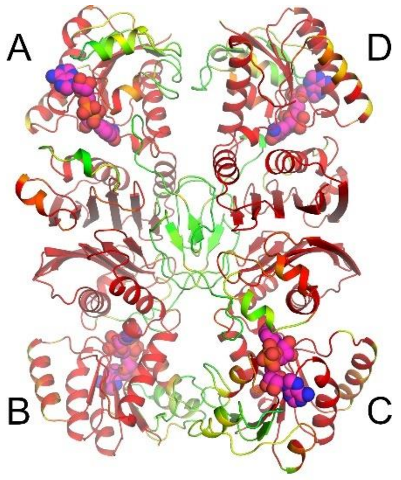

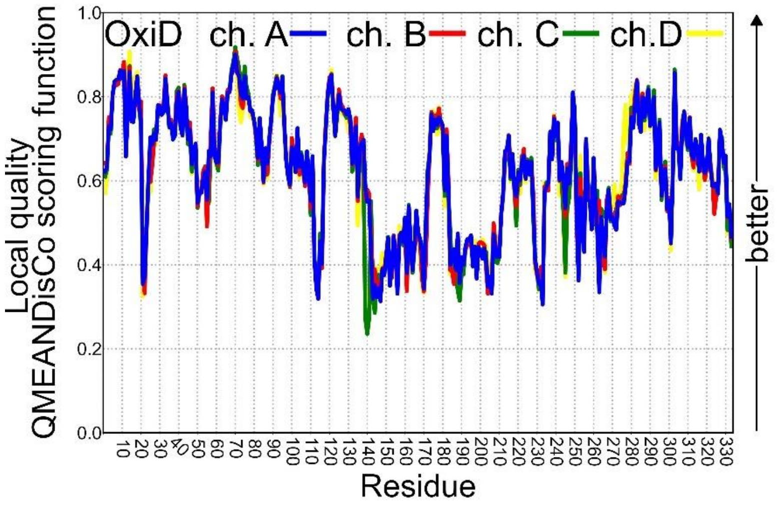

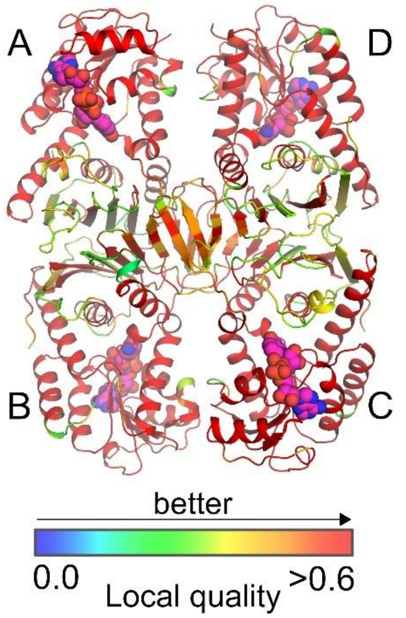

Continued next page

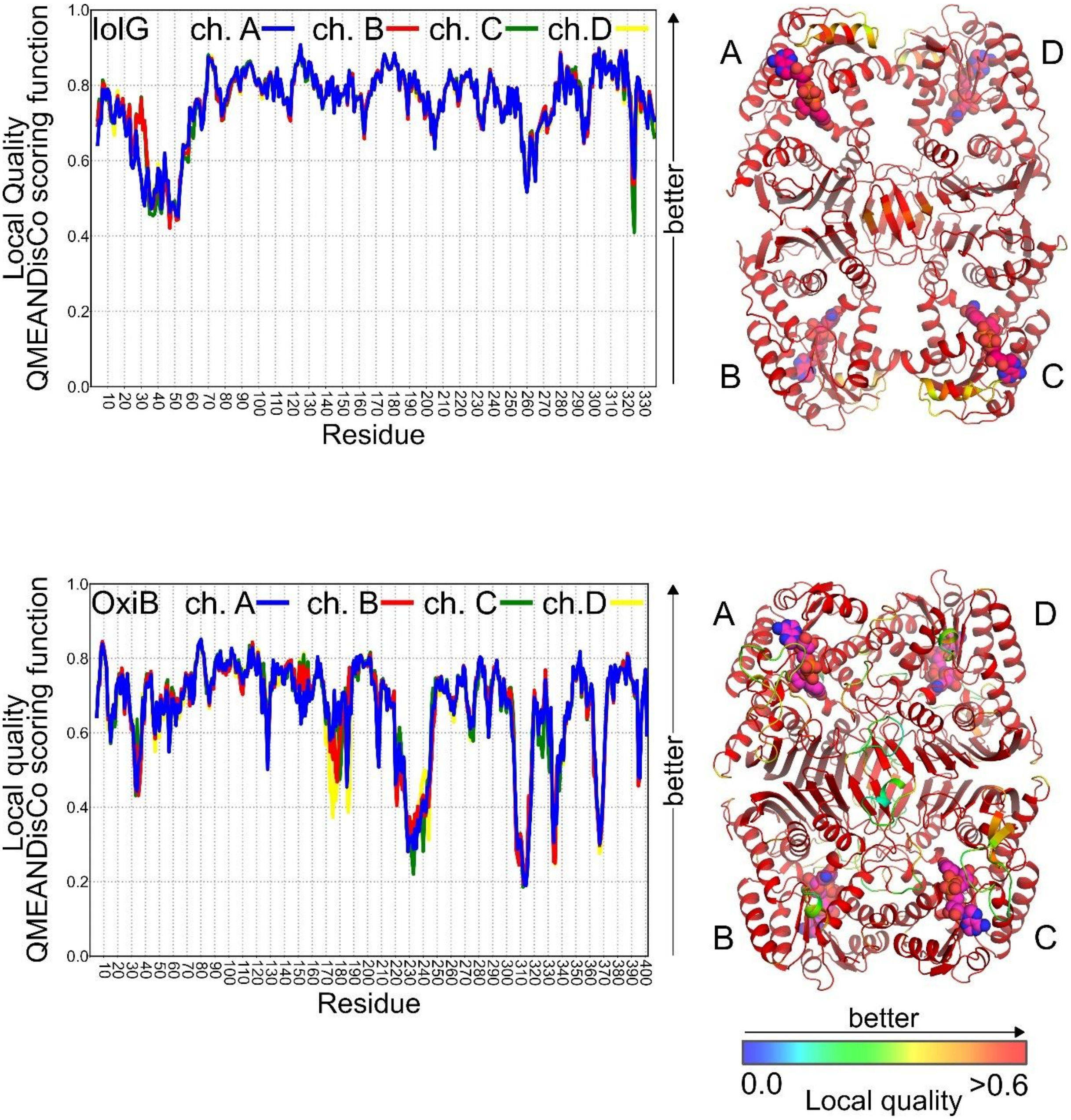

Fig. S6. Quality assessment of modeled IDHs. The per-residue model quality using the QMEANDisCo scoring function (10) is shown for each chain (ch. A, ch. B, ch. C, and ch. D) and mapped onto the modeled IDH structures. Values > 0.6 indicate a good quality. The cofactor positions are highlighted as spheres in magenta.

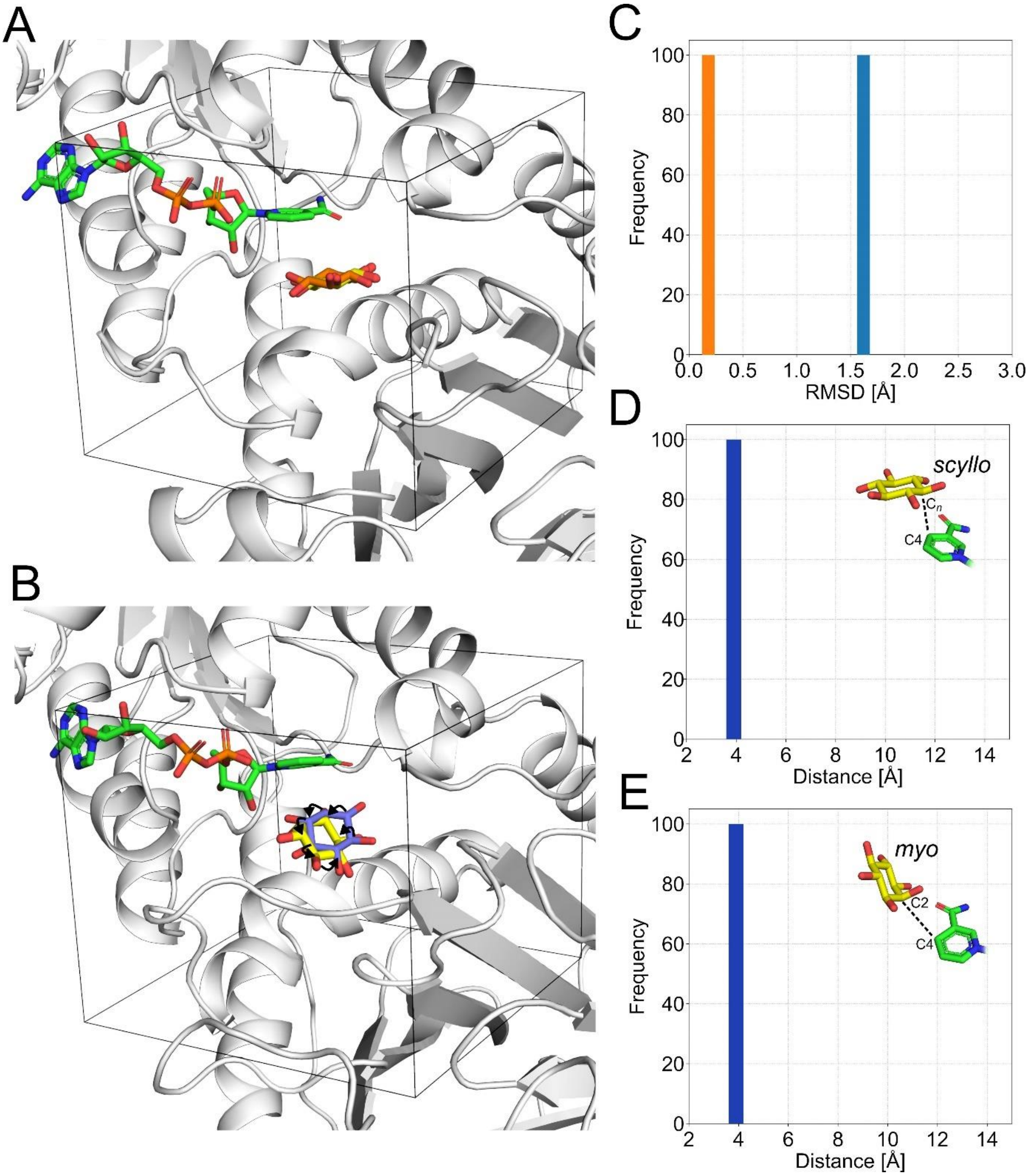

Fig. S7. Redocking of SI and MI. Redocking experiment of (A) SI into *L. casei* IDH2 (PDB id 4N54) and (B) MI into *L. casei* IDH1 (PDB id 4MIO). The SI and MI from the X-ray structures are shown in orange and blue, respectively. All docking poses are colored in yellow. The back box depicts the search space for finding docking poses. In panel B, the docked MI poses are slightly counter-clockwise rotated, indicated by black arrows. (C) Histogram of RMSD values of docked solutions versus the references SI (orange) and MI (blue). Distribution of distances between the reactive carbon of the docked (D) SI (mean 3.9 Å) or (E) MI (mean 4.1 Å) and the C4 carbon of the cofactor's nicotinamide group.

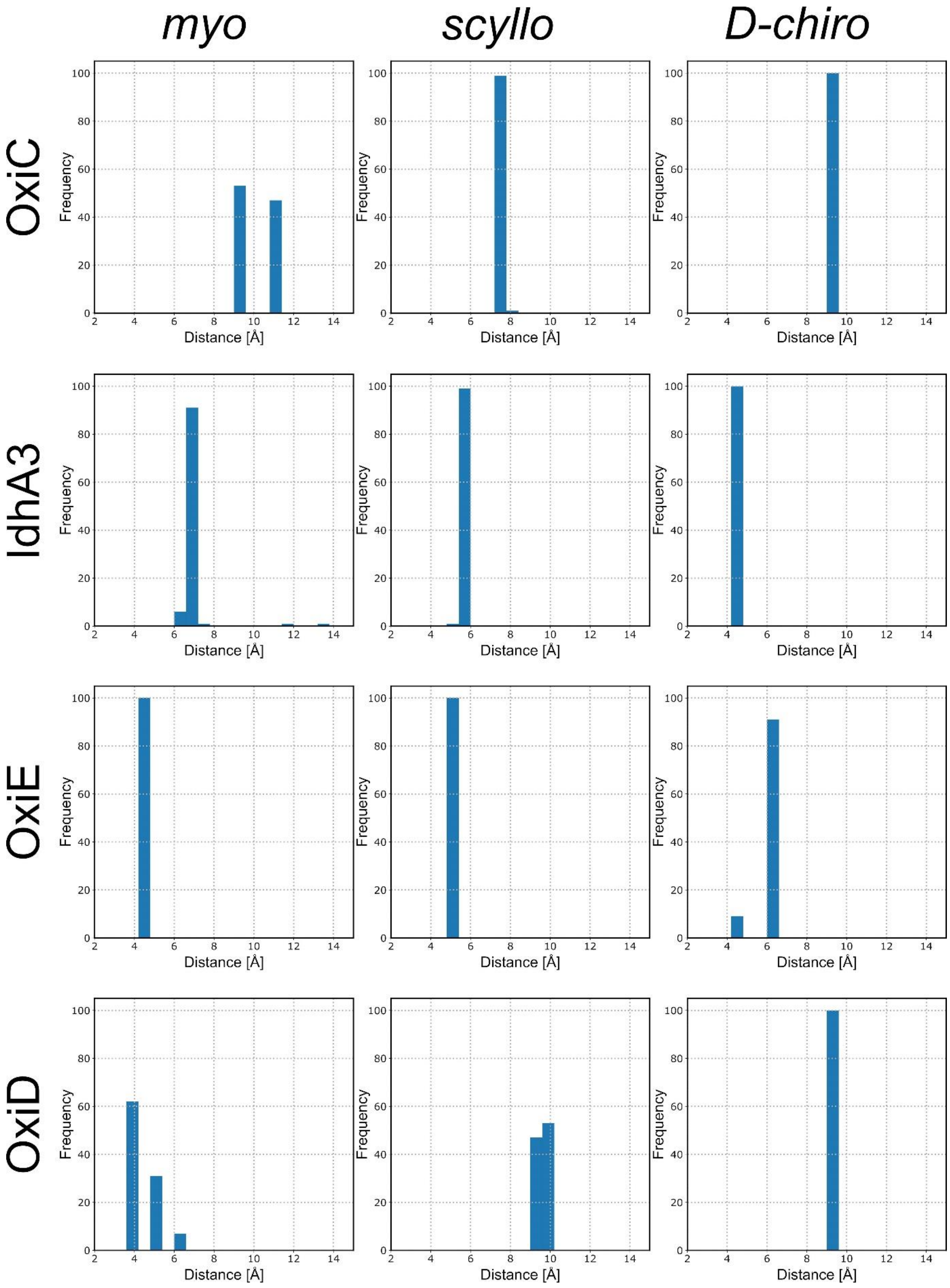

Continued next page

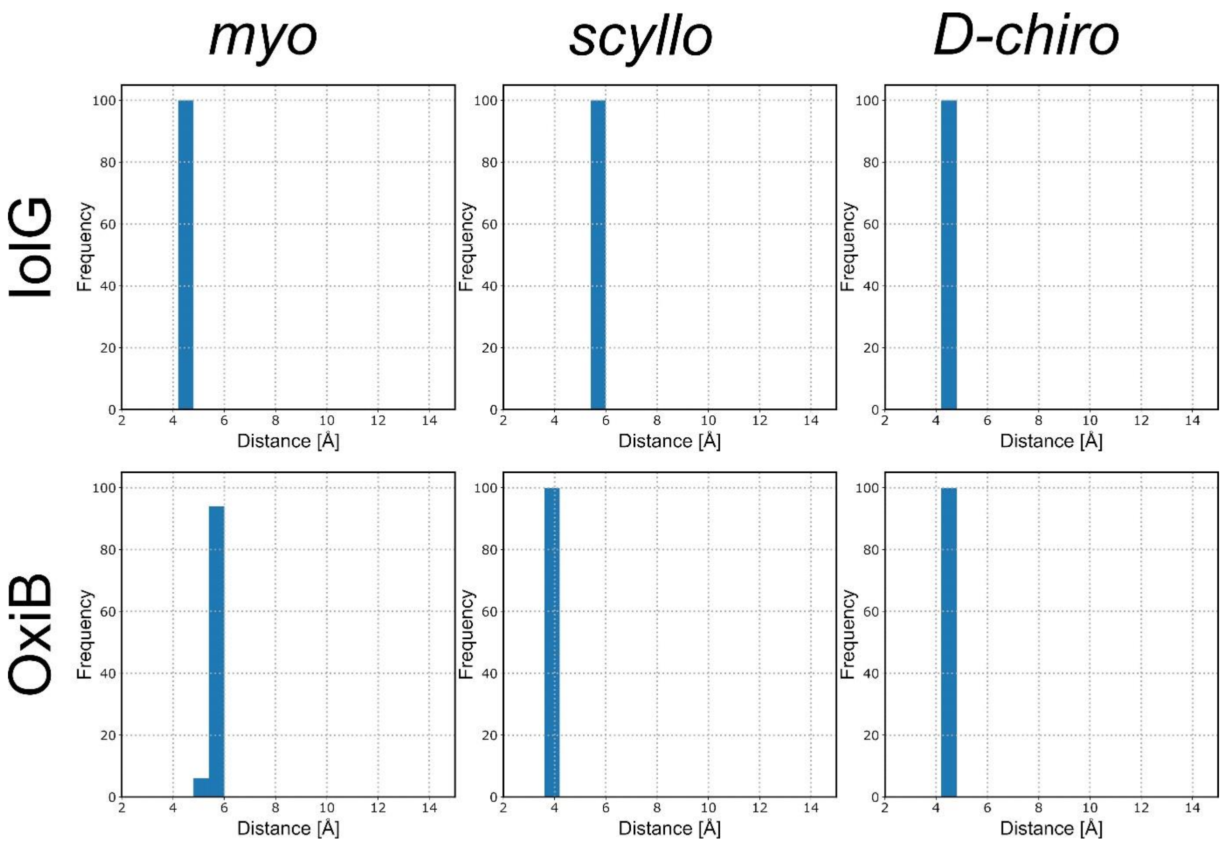

Fig. S8. Distribution of distances between the reactive carbon of the docked MI, SI, or DCI and the C4 carbon of the cofactors nicotinamide group.

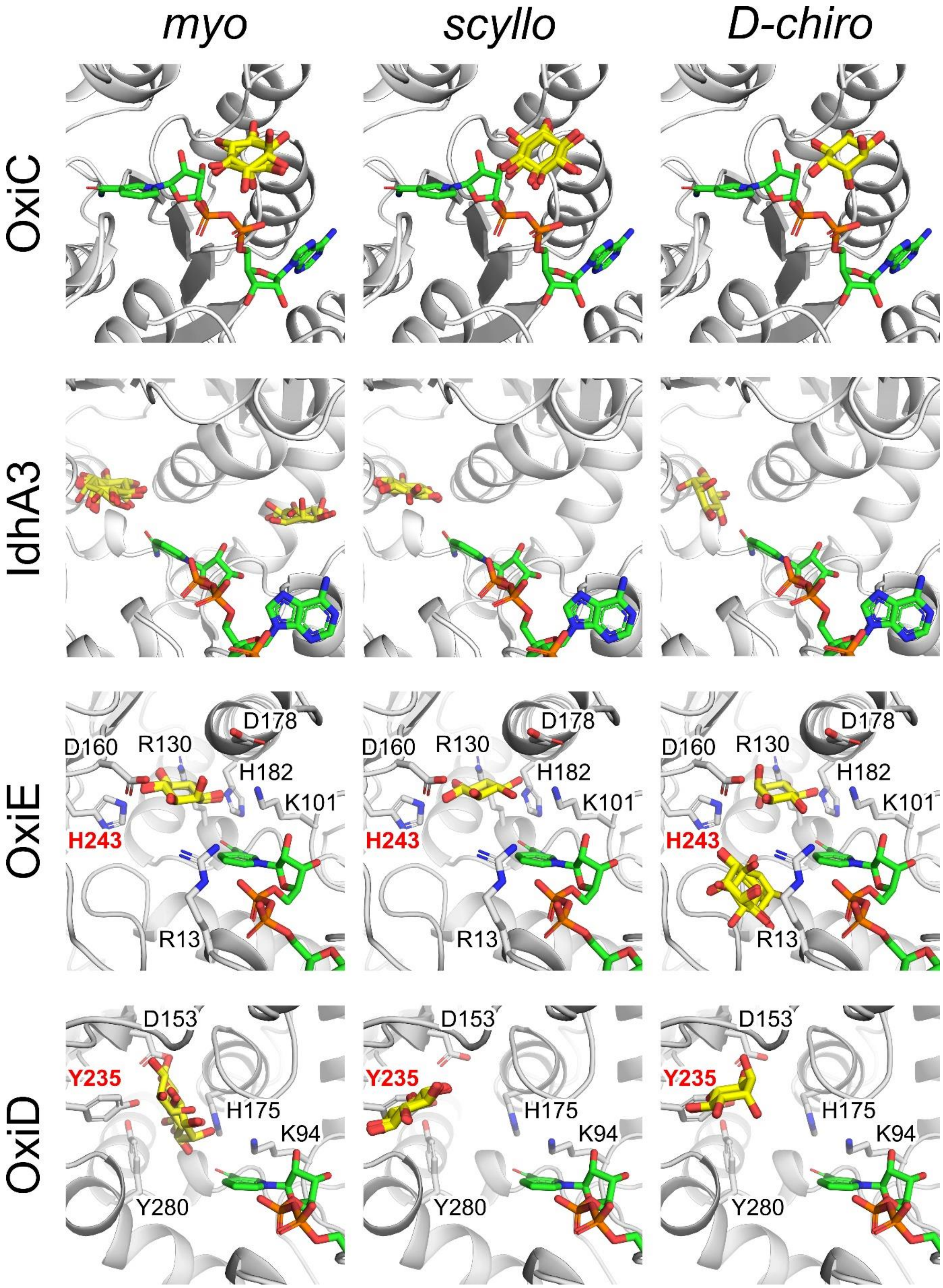

Continued next page

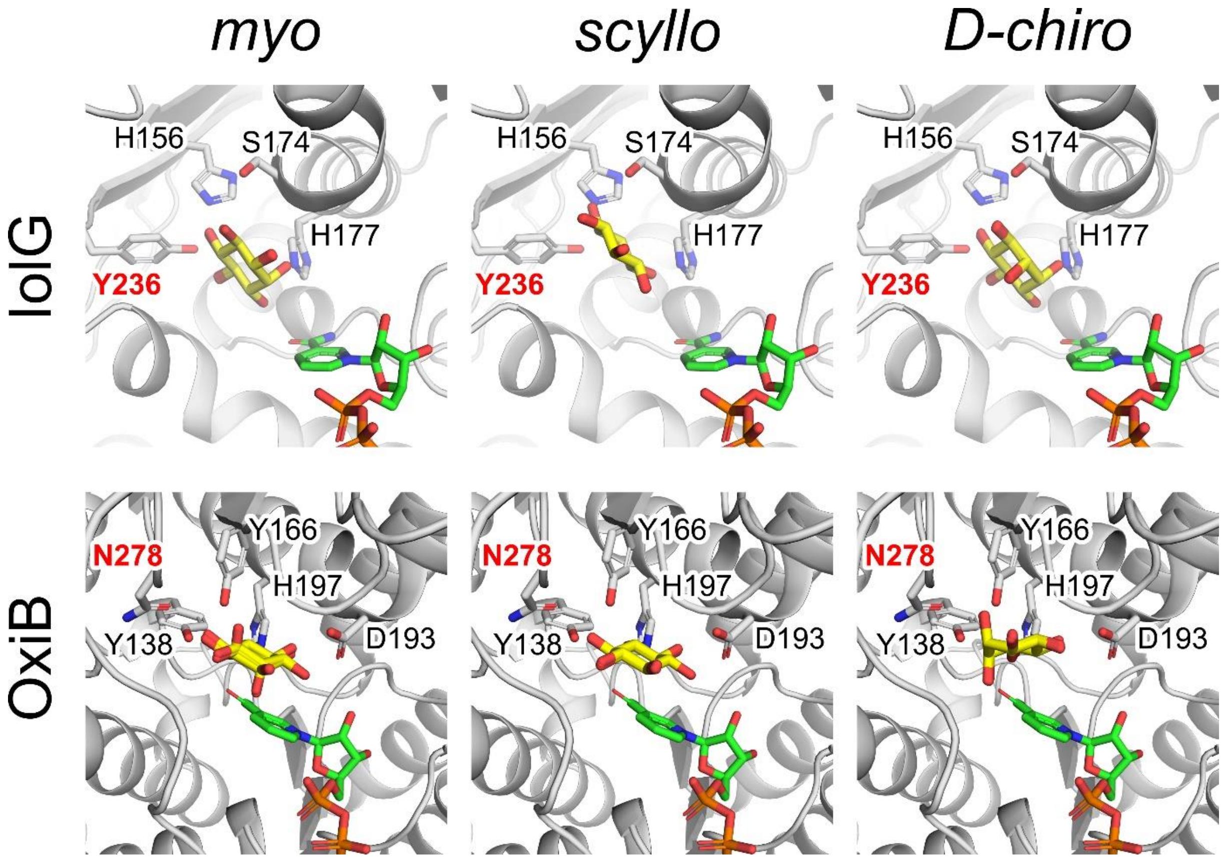

Fig. S9. Docked inositols into IDHs. Docking poses of MI, SI, and DCI into each IDH are shown in yellow, with the cofactor shown in green. If at least one valid docking result is found for the IDH, the residues that potentially form polar interactions with the docked inositol are shown as sticks. The predicted key interactions for IDH selectivity of the inositols (OxiE-H243, OxiD-Y235, IolG-Y236, OxiB-N278) derived from docking studies and the multiple sequence alignment shown (Fig. S5) are labeled in red.

REFERENCES

1. Ramaley R, Fujita Y, Freese E. 1979. Purification and properties of *Bacillus subtilis* inositol dehydrogenase. J Biol Chem 254:7684-7690.
2. Morinaga T, Ashida H, Yoshida K. 2010. Identification of two *scyllo*-inositol dehydrogenases in *Bacillus subtilis*. Microbiology 156:1538-1546.
3. Kang DM, Tanaka K, Takenaka S, Ishikawa S, Yoshida K. 2017. *Bacillus subtilis iolU* encodes an additional $NADP^+$-dependent *scyllo*-inositol dehydrogenase. Biosci Biotechnol Biochem 81:1026-1032.
4. Yoshida K, Sanbongi A, Murakami A, Suzuki H, Takenaka S, Takami H. 2012. Three inositol dehydrogenases involved in utilization and interconversion of inositol stereoisomers in a thermophile, *Geobacillus kaustophilus* HTA426. Microbiology 158:1942-1952.
5. Aamudalapalli HB, Bertwistle D, Palmer DRJ, Sanders DAR. 2018. *myo*-Inositol dehydrogenase and *scyllo*-inositol dehydrogenase from *Lactobacillus casei* BL23 bind their substrates in very different orientations. Biochim Biophys Acta 1866:1115-1124.
6. Rodionova IA, Leyn SA, Burkart MD, Boucher N, Noll KM, Osterman AL, Rodionov DA. 2013. Novel inositol catabolic pathway in *Thermotoga maritima*. Environ Microbiol 15:2254-2266.
7. Kohler PR, Zheng JY, Schoffers E, Rossbach S. 2010. Inositol catabolism, a key pathway in *Sinorhizobium meliloti* for competitive host nodulation. Appl Environ Microbiol 76:7972-7980.
8. Galbraith MP, Feng SF, Borneman J, Triplett EW, de Bruijn FJ, Rossbach S. 1998. A functional *myo*-inositol catabolism pathway is essential for rhizopine utilization by *Sinorhizobium meliloti*. Microbiology 144 2915-2924.
9. Fukano K, Ozawa K, Kokubu M, Shimizu T, Ito S, Sasaki Y, Nakamura A, Yajima S. 2018. Structural basis of L-glucose oxidation by *scyllo*-inositol dehydrogenase: Implications for a novel enzyme subfamily classification. PLoS One 13:e0198010.
10. Studer G, Rempfer C, Waterhouse AM, Gumienny R, Haas J, Schwede T. 2020. QMEANDisCo-distance constraints applied on model quality estimation. Bioinformatics 36:1765-1771.
11. Fasman GD. 2019. CRC Handbook of Biochemistry and Molecular Biology, 3rd edition ed. CRC Press, Taylor and Francis Group, Boca Raton.
